# Supplementary material for: In Silico Binding of 2-Aminocyclobutanones to SARS-CoV-2 Nsp13 Helicase and Demonstration of Antiviral Activity
Source: Int J Mol Sci. 2023 Mar 7;24(6):5120. doi: 10.3390/ijms24065120 (PMC10049026; doi:10.3390/ijms24065120)
Supplement: Supplementary file 1 [file ijms-24-05120-s001.zip › ijms-2206220-supplementary.pdf]

In Silico Binding of 2-Aminocyclobutanones to SARS-CoV-2 Nsp13 Helicase and Demonstration of Antiviral Activity

Table of Contents for Supplementary Materials

Item	Content Detail	Pages
	Table of contents	1-2
Table S1	Full structures of compounds **1a-d** through **10a-d**	3-5
Table S2	Small-molecule SARS-CoV-2 inhibitors targeting Helicase (NSP13)	6-9
procedure	General procedure for the synthesis of N-aryl sulfonamide-amino acids (**12a-c**)	10
procedure	General procedure for the synthesis of amino acid cyclobutanone analogs **1**, **4**, **6**	10-11
procedure	General Procedure for Cbz-protected amino acid cyclobutanone analogs (**2**, **3**, **7**, **9**)	11-12
procedure	(2S)-2-((4-Methylphenyl)sulfonamido)-N-(2-oxocyclobutyl)-3-phenylpropanamide (**1**)	12
procedure	Benzyl ((2R)-1-oxo-1-((2-oxocyclobutyl)amino)-3-phenylpropan-2-yl)carbamate (**2**)	13
procedure	Benzyl ((2S)-3-methyl-1-oxo-1-((2-oxocyclobutyl)amino)butan-2-yl)carbamate (**3**)	13-14
procedure	(2S)-3-(1H-Indol-3-yl)-2-((4-methylphenyl)sulfonamido)-N-(2-oxocyclobutyl)propanamide (4)	14-15
procedure	(S)-N-((1-hydroxycyclopropyl)methyl)-2-((4-methylphenyl)sulfonamido)-3-phenylpropanamide (**5**)	15-16
procedure	(2R)-2-((4-Methylphenyl)sulfonamido)-N-(2-oxocyclobutyl)-3-phenylpropanamide (**6**)	16-17
procedure	Benzyl ((2S)-3-(4-hydroxyphenyl)-1-oxo-1-((2-oxocyclobutyl)amino)propan-2-yl)carbamate (**7**)	17
procedure	(2R)-2-((4-Methoxyphenyl)sulfonamido)-3-methyl-N-(2-oxocyclobutyl)butanamide (**8**)	18-19
procedure	Benzyl ((2S)-1-oxo-1-((2-oxocyclobutyl)amino)-3-phenylpropan-2-yl)carbamate (**9**)	19-20
procedure	(S)-N-(2-hydroxy-2-methylpropyl)-2-((4-methylphenyl)sulfonamido)-3-phenylpropanamide (**10**)	20-21
Figure S1	^{1}H NMR spectrum of (2S)-2-((4-methylphenyl)sulfonamido)-*N*-(2-oxocyclobutyl)-3-phenylpropanamide (**1**)	22
Figure S2	^{1}H NMR spectrum of benzyl ((2R)-1-oxo-1-((2-oxocyclobutyl)amino)-3-phenylpropan-2-yl)carbamate (**2**)	23
Figure S3	^{13}C spectrum of benzyl ((2R)-1-oxo-1-((2-oxocyclobutyl)amino)-3-phenylpropan-2-yl)carbamate (**2**)	24
Figure S4	^{1}H NMR spectrum of benzyl ((2S)-3-methyl-1-oxo-1-((2-oxocyclobutyl)amino)butan-2-yl)carbamate (**3**)	25

Figure S5	^{13}C NMR spectrum of benzyl ((2S)-3-methyl-1-oxo-1-((2-oxocyclobutyl)amino)butan-2-yl)carbamate (**3**)	26
Figure S6	1H spectrum of (2S)-3-(1H-indol-3-yl)-2-((4-methylphenyl)sulfonamido)-N-(2-oxocyclobutyl)propanamide (**4**)	27
Figure S7	^{13}C spectrum of (2*S*)-3-(1*H*-indol-3-yl)-2-((4-methylphenyl)sulfonamido)-*N*-(2-oxocyclobutyl)propanamide (**4**)	28
Figure S8	^{1}H spectrum of (S)-*N*-((1-hydroxycyclopropyl)methyl)-2-((4-methylphenyl)sulfonamido)-3-phenylpropanamide (**5**)	29
Figure S9	^{13}C NMR spectrum of (S)-*N*-((1-hydroxycyclopropyl)methyl)-2-((4-methylphenyl)sulfonamido)-3-phenylpropanamide (**5**)	30
Figure S10	^{1}H NMR spectrum of (2R)-2-((4-methylphenyl)sulfonamido)-*N*-(2-oxocyclobutyl)-3-phenylpropanamide (**6**)	31
Figure S11	^{13}C spectrum of (2R)-2-((4-methylphenyl)sulfonamido)-*N*-(2-oxocyclobutyl)-3-phenylpropanamide (**6**)	32
Figure S12	^{1}H NMR spectrum of benzyl ((2S)-3-(4-hydroxyphenyl)-1-oxo-1-((2-oxocyclobutyl)amino)propan-2-yl)carbamate (**7**)	33
Figure S13	^{13}C NMR spectrum of benzyl ((2S)-3-(4-hydroxyphenyl)-1-oxo-1-((2-oxocyclobutyl)amino)propan-2-yl)carbamate (**7**)	34
Figure S14	: ^{1}H spectrum of (2R)-2-((4-methoxyphenyl)sulfonamido)-3-methyl-*N*-(2-oxocyclobutyl)butanamide (**8**)	35
Figure S15	^{13}C spectrum of (2R)-2-((4-methoxyphenyl)sulfonamido)-3-methyl-*N*-(2-oxocyclobutyl)butanamide (**8**)	36
Figure S16	^{1}H NMR spectrum of benzyl ((2S)-1-oxo-1-((2-oxocyclobutyl)amino)-3-phenylpropan-2-yl)carbamate (**9**)	37
Figure S17	^{13}C NMR spectrum of benzyl ((2S)-1-oxo-1-((2-oxocyclobutyl)amino)-3-phenylpropan-2-yl)carbamate (**9**)	38
Figure S18	^{1}H NMR spectrum of (S)-*N*-(2-hydroxy-2-methylpropyl)-2-((4-methylphenyl)sulfonamido)-3-phenylpropanamide (**10**)	39
Figure S19	^{13}C NMR spectrum of (S)-*N*-(2-hydroxy-2-methylpropyl)-2-((4-methylphenyl)sulfonamido)-3-phenylpropanamide (**10**)	40

Table S1. Docking parameters and calculated energies of amino acid-derived cyclobutanone hits against SARS-CoV-2 helicase.

#	Structure	Glide Score	dG Bind Energy	LogP[b]	mp (°C)	Cell[c] 5 µM	Cell[d] 50 µM
**1**	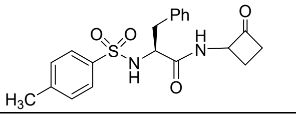			2.6	177-180	39.3	49.8
**1a**	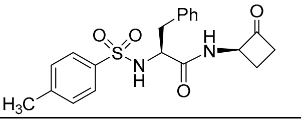	-4.012242	-29.14638				

**1b**	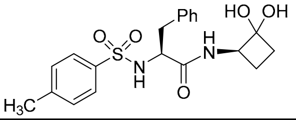	-6.751353	-28.84719				
**1c**	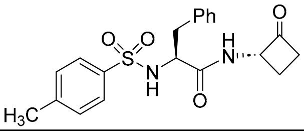	-3.765231	-27.9652				
**1d**	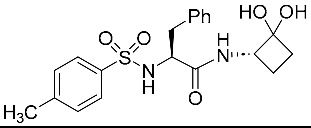	-4.447791	-20.97472				
**2**	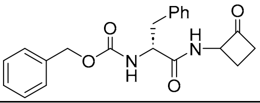			2.9	98-100	24.2	34.7
**2a**	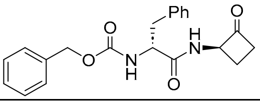	-6.161241	-34.03249				
**2b**	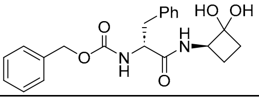	-4.512567	-42.28711				
**2c**	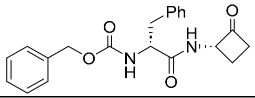	-3.59575	-25.1313				
**2d**	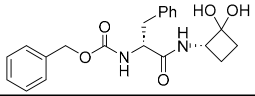	-4.938537	-38.28715				
**3**	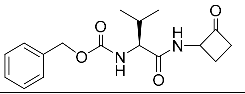			2.1	140-144	37.9	54.9
**3a**	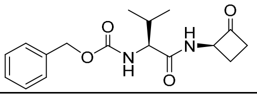	-4.096235	-47.9141				
**3b**	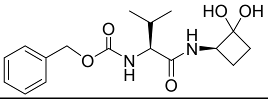	-5.27515	-55.17853				
**3c**	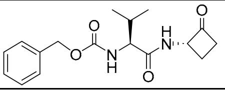	-3.818151	-37.84926				
**3d**	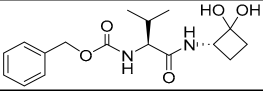	-6.073736	-56.36429				
**4**	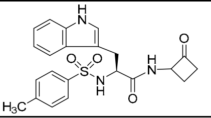			2.7	200-201	54.3	70.9
**4d**	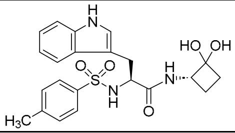	-5.859944	-37.83419				

**5**	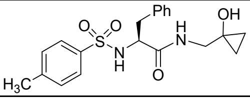	-5.79524	-28.19968	2.2	131-132	30.0	30.7
**6**	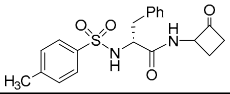			2.6	190-192	26.6	35.9
**6a**	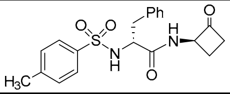	-3.5261	-21.16836				
**6b**	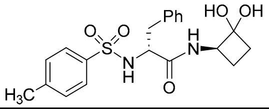	-5.71961	-24.16271				
**6c**	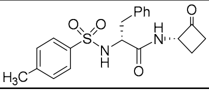	-3.891619	-24.29386				
**6d**	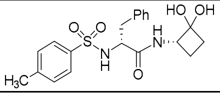	-4.905462	-23.3425				
**7**	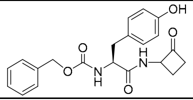			2.5	154-155	-2	28.1
**7a**	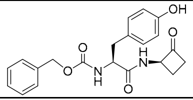	-4.050345	-39.41216				
**7b**	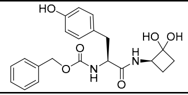	-5.690338	-34.44169				
**7c**	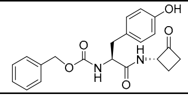	-4.872337	-36.31399				
**7d**	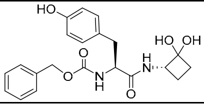	-5.418732	-30.65196				
**8**	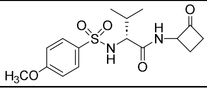			1.3	179-181	32.0	49.1
**8b**	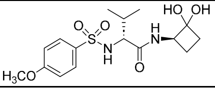	-5.27903	-18.91634				
**9**	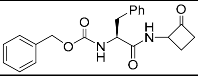			2.9	100-101	41.6	57.5
**9a**	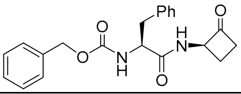	-4.949141	-31.68122				
**9c**	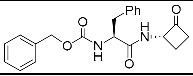	-5.038913	-38.05829				
**10**	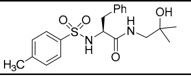	-3.923168	-29.98338	2.6	131-133	36.7	59.2

[a]Compound letters indicate chirality of carbon bearing the cyclobutanone alpha-amino moiety as well as carbonyl versus hydrated forms, where **a** = (R)-stereochemistry and cyclobutanone carbonyl; **b** = (R)-stereochemistry and ketone hydrate; **c** = (S)-stereochemistry and cyclobutanone ketone; **d** = (S)-stereochemistry and ketone hydrate. Full structures are included in the Supporting Information as Tables of content. [b]LogP is the Molecular Networks

calculated cLogP; [c]Cell Survival following SARS-CoV-2 Challenge at 5 µM; [d]Cell Survival following SARS-CoV-2 Challenge at 50 µM.

Table S2. Small-molecule SARS-CoV-2 inhibitors targeting Helicase (NSP13)

#	Class	Structure	Test model	Enzme Assay IC_{50} (μM)	Antiviral activity IC_{50}	Tox CC_{50} (μmol/L)	Clinical or Pre-clin info	Ref
**1**	Aryl diketoacids	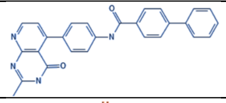	Enzyme Assay	6 ± 0.5	n/a	n/a	n/a	Ref[1]
**2**	Bananins	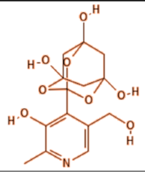	SARS-CoV Helicase enzyme	2.3 μM	n/a	n/a	n/a	Ref[2]
**3**	2,6-Bis-arylmethyloxy-5-hydroxy-chromones	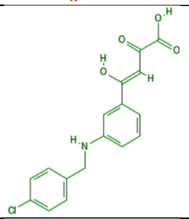	Enzyme and in vitro assay	4 μM	4 μM	n/a	n/a	Ref[3]
**4**	SSYA10-001	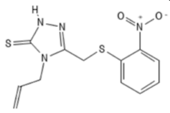	In vitro	n/a	n/a	80.5	n/a	Ref[4]
**5**	Rosmanol	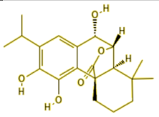	Enzyme assay	8.93	n/a	n/a	n/a	Ref[5]
**6**	Katacine	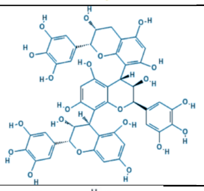	Enzyme assay	5.98	n/a	n/a	n/a	Ref[5]
**7**	UMI-77	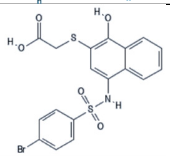	Enzyme assay	4.38	n/a	n/a	n/a	Ref[5]
**8**	Zinc Orotate	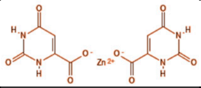	Enzyme assay	4.12	n/a	n/a	n/a	Ref[5]
**9**	MC-Val-Cit-PABC-PNP (peptide mimentic)	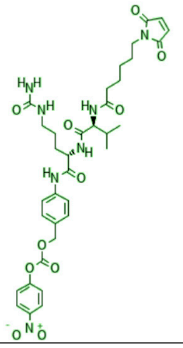	Enzyme assay	2.45	n/a	n/a	n/a	Ref[5]

**10**	Silver sulfadiazine	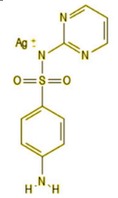	Enzyme assay	2.11	n/a	n/a	n/a	Ref[5]
**11**	(−)-Gallocatechin gallate	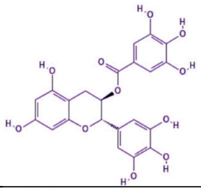	Enzyme assay	1.34	n/a	n/a	n/a	Ref[5]
**12**	Tannic acid	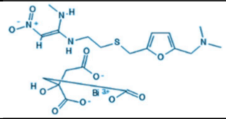	Enzyme assay	1.25	n/a	n/a	n/a	Ref[5]
**13**	Dyngo-4a	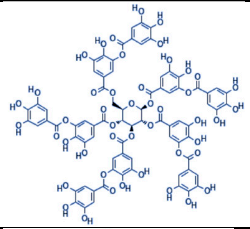	Enzyme assay	0.63	n/a	n/a	n/a	Ref[5]
**14**	Rhodiosin	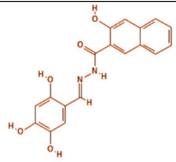	Enzyme assay	0.48	n/a	n/a	n/a	Ref[5]
**15**	Punicalagin	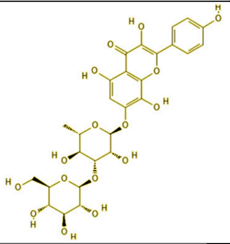	Enzyme and in vitro assay	0.43	347 nM	33.92	n/a	Ref[5]
**16**	Grazoprevir	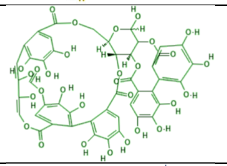	in vitro assay	n/a	1.41	32 .71 ± 0.94 µM	n/a	Ref[6]
**17**	Zafirlukast	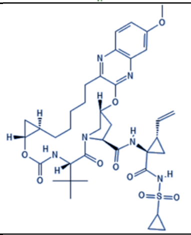	Enzyme assay	16.3	n/a	n/a	n/a	Ref[7]
**18**	licoflavone C	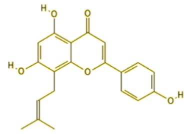	Enzyme assay	1.34 ± 0.31	n/a	n/a	n/a	Ref[8]

**19**	FPA124	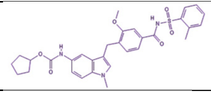	Enzyme assay	8.5	14.0 µM	n/a	n/a	Ref[9]
**20**	Disulfiram	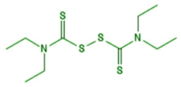	Enzyme and in vitro assay	0.41	3.180 µM	n/a	n/a	Ref[10]
**21**	Ranitidine bismuth citrate	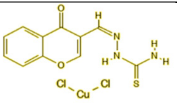	In vitro & in vivo (Sirian hamster)	0.69	n/a	3,254 ± 21 µM	1 to 1.5 log viral load redn in Animal lungs & nasal turbin-ate	Ref[11]

References for Table S2

1. Yazdi, A. K.; Pakarian, P.; Perveen, S.; Hajian, T.; Santhakumar, V.; Bolotokova, A.; Li, F.; Vedadi, M. Kinetic Characterization of SARS-CoV-2 nsp13 ATPase Activity and Discovery of Small-Molecule Inhibitors. *ACS Infectious Diseases* **2022**, *8,* 1533-1542.

2. Tanner, J. A.; Zheng, B.; Zhou, J.; Watt, R. M.; Jiang, J.; Wong, K.; Lin, Y.; Lu, L.; He, M.; Kung, H. The adamantane-derived bananins are potent inhibitors of the helicase activities and replication of SARS coronavirus. *Chem. Biol.* **2005**, *12,* 303-311.

3. Kim, M. K.; Yu, M.; Park, H. R.; Kim, K. B.; Lee, C.; Cho, S. Y.; Kang, J.; Yoon, H.; Kim, D.; Choo, H. 2, 6-Bis-arylmethyloxy-5-hydroxychromones with antiviral activity against both hepatitis C virus (HCV) and SARS-associated coronavirus (SCV). *Eur. J. Med. Chem.* **2011**, *46,* 5698-5704.

4. Canal, B.; McClure, A. W.; Curran, J. F.; Wu, M.; Ulferts, R.; Weissmann, F.; Zeng, J.; Bertolin, A. P.; Milligan, J. C.; Basu, S. Identifying SARS-CoV-2 antiviral compounds by screening for small molecule inhibitors of nsp14/nsp10 exoribonuclease. *Biochem. J.* **2021**, *478,* 2445-2464.

5. Lu, L.; Peng, Y.; Yao, H.; Wang, Y.; Li, J.; Yang, Y.; Lin, Z. Punicalagin as an allosteric NSP13 helicase inhibitor potently suppresses SARS-CoV-2 replication in vitro. *Antiviral Res.* **2022**, *206,* 105389.

6. Muturi, E.; Hong, W.; Li, J.; Yang, W.; He, J.; Wei, H.; Yang, H. Effects of simeprevir on the replication of SARS-CoV-2 in vitro and in transgenic hACE2 mice. *Int. J. Antimicrob. Agents* **2022**, *59,* 106499.

7. Mehyar, N.; Mashhour, A.; Islam, I.; Alhadrami, H.; Tolah, A.; Alghanem, B.; Alkhaldi, S.; Somaie, B.; Al Ghobain, M.; Alobaida, Y. Discovery of Zafirlukast as a novel SARS-CoV-2 helicase inhibitor using in silico modelling and a FRET-based assay. *SAR QSAR Environ. Res.* **2021**, *32,* 963-983.

8. Corona, A.; Wycisk, K.; Talarico, C.; Manelfi, C.; Milia, J.; Cannalire, R.; Esposito, F.; Gribbon, P.; Zaliani, A.; Iaconis, D. Natural compounds inhibit SARS-CoV-2 nsp13 unwinding and ATPase enzyme activities. *ACS pharmacology & translational science* **2022**, *5,* 226-239.

9. Zeng, J.; Weissmann, F.; Bertolin, A. P.; Posse, V.; Canal, B.; Ulferts, R.; Wu, M.; Harvey, R.; Hussain, S.; Milligan, J. C. Identifying SARS-CoV-2 antiviral compounds by screening for small molecule inhibitors of nsp13 helicase. *Biochem. J.* **2021**, *478,* 2405-2423.

10. Chen, T.; Fei, C.; Chen, Y.; Sargsyan, K.; Chang, C.; Yuan, H. S.; Lim, C. Synergistic inhibition of SARS-CoV-2 replication using disulfiram/ebselen and remdesivir. *ACS Pharmacology & Translational Science* **2021**, *4,* 898-907.

11. Yuan, S.; Wang, R.; Chan, J. F.; Zhang, A. J.; Cheng, T.; Chik, K. K.; Ye, Z.; Wang, S.; Lee, A. C.; Jin, L. Metallodrug ranitidine bismuth citrate suppresses SARS-CoV-2 replication and relieves virus-associated pneumonia in Syrian hamsters. *Nature microbiology* **2020**, *5,* 1439-1448.

Experimental

General procedure for the synthesis of *N*-aryl sulfonamide-amino acids (12a-c)

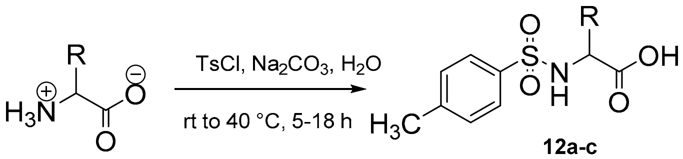

Following the general method reported by Misra and co-workers,[1] to a vigorously stirring solution of the amino acid (1.21 mmol) in DI water (1.5 mL), sodium carbonate (179.8 mg, 1.45 mmol) was added. Once all of the solutes were dissolved, the solution was cooled to 0°C and the respective aryl sulfonyl chloride (1.45 mmol) was added to the stirring reaction mixture in four portions over 1 h. Then the reaction was allowed to warm up to rt and the slurry was stirred at rt for 4 h or until completion. The reaction progress was monitored by TLC (methanol/methylene chloride = 10/90) Once completed, the mixture was acidified using 2 N HCl until the pH of the solution was reduced to pH = 2. Crystals formed during the acidification were filtered and washed with a pH 2.2 buffer. Final pure products (**12a-c**) were dried under a high vacuum. Both proton and carbon NMR spectra of pure products **12a-c** matched the reported characterization data as indicated.

General procedure for the synthesis of amino acid cyclobutanone analogs 1, 4, 6.

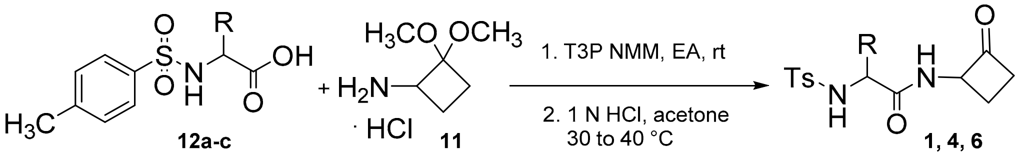

To a solution of substituted *N*-benzene sulfonamide amino acids **12a-c** (1 eq) and 2,2-dimethoxycyclobutan-1-aminium chloride[2] (**11**) (1.2 eq) in ethyl acetate (0.2 M), *N*-methyl

morpholine (4.0 eq) was added. Then propylphosphonic anhydride (T3P, 2.5 eq) was added to the above mixture as a solution in ethyl acetate (purchased as ≥ 50 weight % in EA) The reaction mixture was stirred under N_2 at rt to 40°C for 18 hours or until complete consumption of protected carboxylic acid intermediates determined by HPLC analysis. The reaction was quenched by adding water (3 mL) and then the organic product was extracted using ethyl acetate (3 x 3 mL) The combined organic layers were washed successively with water (3 x 3 mL) and 1N HCl (3 mL) and then dried over Na_2SO_4. The solvent was removed by evaporation under reduced pressure providing the corresponding acetal intermediate. Without further purification crude acetal (1 eq, final concentration was 0.1 M) was subjected to hydrolysis conditions, wherein the crude mixture was dissolved in acetone, water (10% v/v), and 1N HCl (30% v/v) Then the reaction mixture was stirred at 40°C overnight with periodic HPLC monitoring. Upon completion, the organic product was extracted using ethyl acetate (3 x 3 mL) or methylene chloride for highly water-soluble analogs and the combined organic layers were dried over Na_2SO_4. The solvent was evaporated under reduced pressure and the crude mixture was purified by column chromatography to afford the corresponding amino acid-derived cyclobutanone (**1, 4, 6**)

General Procedure for Cbz-protected amino acid cyclobutanone analogs (2, 3, 7, 9)

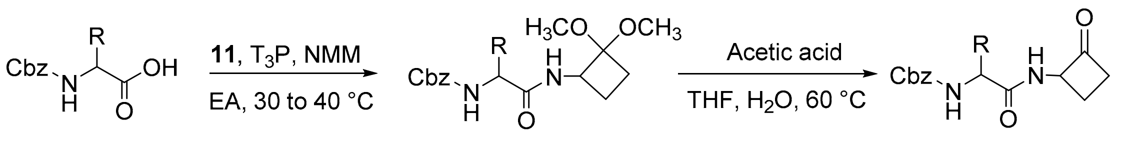

13a-d, **a**: D-Phe, **b**: L-Val, **c**: L-Tyr, **d**: L-Phe

2, **3**, **7**, **9**

Cbz-protected amino acids (Phe, Try, Val, 50 mg) were coupled with 2-aminocyclobutanone synthon **11** following the general T3P coupling reaction procedure. The resulting acetal

intermediates (**13a-d**) were subjected to hydrolysis without further purification. To a solution crude acetal product (1 eq, concentration = 0.1 M) in THF (40% v/v), water (20% v/v) and acetic acid were added, and the reaction mixture was stirred at 60 °C overnight or until deemed complete by HPLC. Upon completion, excess acetic acid was neutralized by adding a solution of saturated Na_2CO_3 until a pH of 8 was achieved and the organic product was extracted with methylene chloride (3 x 3 mL) The combined organic layers were dried over Na_2SO_4 and the solvent was evaporated under reduced pressure. The crude mixture was purified by column chromatography to afford the corresponding Cbz-amino acid-cyclobutanone (**2**, **3**, **7**, **9**).

(2*S*)-2-((4-Methylphenyl)sulfonamido)-*N*-(2-oxocyclobutyl)-3-phenylpropanamide (1)

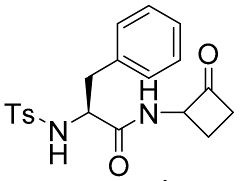

1

The *N*-Ts-carboxylic intermediate **12a** (50.0 mg, 0.129 mmol) was used in the reaction. The crude mixture of **1** was purified by column chromatography eluting with a gradient of ethyl acetate/hexane (20/80) and ethyl acetate/hexane (40/60) followed by recrystallization from hot EA to afford compound **1** (9.8 mg, 20%) as white crystalline solid. ^{1}H NMR (500 MHz, $CDCl_3$) δ 7.57 – 7.44 (m, 2H), 7.25 – 7.15 (m, 5H), 6.96 – 6.87 (m, 2H), 6.72 (d, *J* = 8.3 Hz, 1H), 4.97 (dtt, *J* = 10.1, 8.2, 2.1 Hz, 1H), 4.85 (d, *J* = 6.6 Hz, 1H), 3.85 (dt, *J* = 7.3, 6.4 Hz, 1H), 3.01 – 2.89 (m, 3H), 2.88 (dddd, *J* = 17.4, 9.9, 4.7, 2.3 Hz, 1H), 2.43 (s, 3H), 2.46 – 2.35 (m, 1H), 1.86 (dtd, *J* = 11.1, 9.7, 8.3 Hz, 1H) HRMS (ESI): Calcd for (MH^+) $C_{20}H_{23}N_2O_4S$: 387.1373, found 387.1366.

Benzyl ((2R)-1-oxo-1-((2-oxocyclobutyl)amino)-3-phenylpropan-2-yl)carbamate (2)

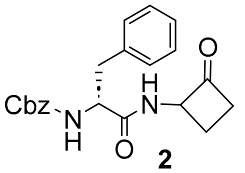

2

The crude mixture of **2** was purified by column chromatography eluting with a gradient of ethyl acetate/hexane (20/80) and ethyl acetate/hexane (30/70) Appropriate fractions were combined and concentrated to give white solid (93% HPLC purity) which then subjected to recrystallization. The product was dissolved in minimum amount of methylene chloride and hexane was added dropwise until cloudiness was observed. Colorless crystals were formed providing compound **2** (16.1 mg, 26%): mp 98-100 °C. ^{1}H NMR (500 MHz, $CDCl_3$) δ 7.41 – 7.24 (m, 8H), 7.21 (t, *J* = 6.0 Hz, 2H), 6.35 (d, *J* = 7.6 Hz, 0.5H), 6.20 (d, *J* = 8.0 Hz, 0.5H), 5.32 (s, 0.5H), 5.27 (s, 0.5H), 5.10 (s, 2H), 4.92 (q, *J* = 9.0 Hz, 0.5H), 4.81 (q, *J* = 8.7 Hz, 0.5H), 4.42 (q, *J* = 7.3 Hz, 1H), 3.17 – 3.11 (m, 1H), 3.06 (td, *J* = 13.5, 12.9, 7.6 Hz, 1H), 2.99 – 2.85 (m, 2H), 2.39 (dq, *J* = 15.8, 9.7 Hz, 1H), 2.06 - 2.03 (m, 0.5H), 1.88 – 1.81 (m, 0.5H) ^{13}C NMR (126 MHz, $CDCl_3$) δ 204.33, 170.65, 170.49, 155.97, 136.02, 136.02, 129.39, 129.37, 128.84, 128.59, 128.30, 128.29, 128.09, 127.22, 67.23, 64.05, 63.76, 56.03, 42.21, 42.12, 38.56, 38.19, 19.42, 19.22.

Benzyl ((2S)-3-methyl-1-oxo-1-((2-oxocyclobutyl)amino)butan-2-yl)carbamate (3)

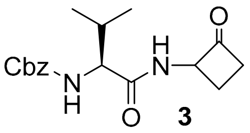

3

The crude mixture of **3** was purified by column chromatography eluting with a gradient of ethyl acetate/hexane (20/80) to (30/70) Appropriate fractions were combined and

concentrated to give a white solid which then subjected to recrystallization. The product was dissolved in a minimum amount of methylene chloride and hexane was added dropwise until cloudiness was observed. Over 24 hours, needle-like crystals formed providing compound **3** (21.3 mg, 34%) ^{1}H NMR (500 MHz, $CDCl_3$) δ 7.38 (s, 3H), 7.43 – 7.32 (m, 2H), 6.44 – 6.39 (d, 1H), 5.26 (d, *J* = 8.9 Hz, 1H), 5.14 (s, 2H), 4.91 (dt, *J* = 10.1, 7.9 Hz, 1H), 4.01 (dd, *J* = 8.8, 6.0 Hz, 1H), 2.98 (t, *J* = 8.8 Hz, 2H), 2.46 (dd, *J* = 13.3, 5.3 Hz, 1H), 2.18 (h, *J* = 6.8 Hz, 1H), 2.13 – 2.06 (m, 1H), 1.00 (d, *J* = 6.8 Hz, 3H), 0.95 (d, *J* = 6.8 Hz, 3H) ^{13}C NMR (126 MHz, $CDCl_3$) δ 204.44, 170.99, 152.17, 136.09, 128.61, 128.31, 128.13, 67.27, 64.17, 60.03, 42.28, 30.79, 29.71, 19.48, 19.18. HRMS (ESI): Calcd for (MH^+) $C_{17}H_{23}N_2O_4$: 319.1652, found 319.1641.

(2*S*)-3-(1*H*-Indol-3-yl)-2-((4-methylphenyl)sulfonamido)-*N*-(2-oxocyclobutyl)propanamide (4)

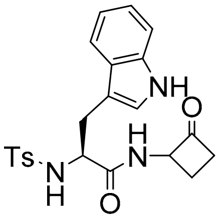

4

The *N*-Ts-carboxylic intermediate **12b** (50.0 mg, 0.139 mmol) was used in the reaction. The crude mixture of **4** was purified by column chromatography on a Teledyne Isco Rf Flash chromatography unit eluting with a gradient of ethyl acetate/hexane (20/80), ethyl acetate/hexane (30/70), and ethyl acetate/hexane (40/60) to afford compound **4** (12.4 mg, 21%) as an off-white solid: mp 200-201 °C. ^{1}H NMR (500 MHz, acetonitrile-d_3) δ 9.07 (NH, s, 1H), 7.48 – 7.41 (m, 2H), 7.41 – 7.32 (m, 2H), 7.16 – 7.09 (m, 3H), 7.10 (NH, d, *J* = 7.5 Hz,

1H), 7.07 – 6.96 (m, 2H), 5.82 (NH, s, 1H), 4.68 (dtt, *J* = 10.2, 8.0, 2.2 Hz, 1H), 3.90 (dd, *J* = 8.1, 5.7 Hz, 1H), 3.14 – 3.06 (m, 1H), 2.95 – 2.73 (m, 3H), 2.35 (s, 3H), 2.21 – 2.10 (m, 1H), 1.82 (tt, *J* = 10.5, 8.6 Hz, 1H) ^{13}C NMR (126 MHz, CD_3CN) δ 205.61, 170.72, 143.47, 136.61, 136.41, 129.35, 127.10, 126.57, 126.51, 124.06, 121.41, 118.88, 111.33, 108.97, 63.62, 63.60, 57.04, 41.40, 41.34, 28.72, 28.58, 20.60, 20.58, 18.18, 18.11. HRMS (ESI): Calcd for (MH^+) $C_{22}H_{24}N_3O_4S$: 426.1482, found 426.1472.

(*S*)-*N*-((1-hydroxycyclopropyl)methyl)-2-((4-methylphenyl)sulfonamido)-3-phenylpropanamide (5)

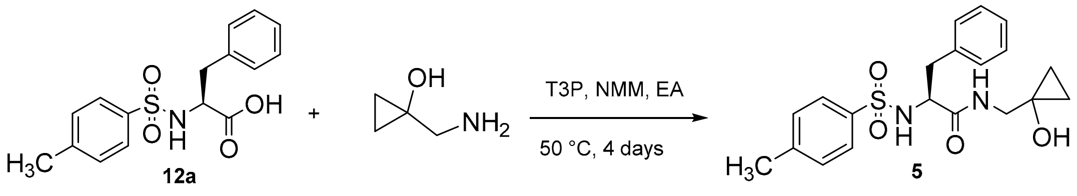

Ts-L-phenylalanine **12a** (50 mg, 0.129 mmol) was reacted with 1-(aminomethyl)cyclopropanol (14.4 µL, 0.155 mmol) following the general T3P coupling procedure and stirred at 50 °C for 4 days. The crude mixture of **5** was purified by column chromatography on a Teledyne Isco Rf Flash chromatography unit eluting with a gradient of ethyl acetate/hexane (20/80) and ethyl acetate/hexane (30/70) Appropriate fractions were combined and concentrated to give a white solid which was then subjected to recrystallization. The product was dissolved in a minimum amount of hot methylene chloride and hexane was added dropwise until cloudiness was observed. While cooling needle-like crystals formed providing compound **5** (13.6 mg, 27%) mp 131-133 °C. ^1H NMR (500 MHz, $CDCl_3$) δ 7.51 (dd, *J* = 8.1, 2.4 Hz, 2H), 7.25 (d, *J* = 13.9 Hz, 1H), 7.21 (dd, *J* =

12.2, 7.2 Hz, 5H), 6.94 (d, *J* = 7.1 Hz, 2H), 6.75 (NH, d, *J* = 6.3 Hz, 1H), 4.96 (NH, d, *J* = 5.7 Hz, 1H), 3.86 – 3.80 (m, 1H), 3.39 (ddd, *J* = 14.3, 6.8, 2.4 Hz, 1H), 3.30 – 3.22 (m, 1H), 3.20 (OH, s, 1H), 2.95 (ttd, *J* = 14.1, 10.3, 8.5, 4.4 Hz, 2H), 2.43 (d, *J* = 2.4 Hz, 3H), 0.79 (s, 2H), 0.64 – 0.56 (m, 1H), 0.56 – 0.50 (m, 1H) ^{13}C NMR (126 MHz, $CDCl_3$) δ 171.13, 144.15, 135.17, 135.03, 129.88, 129.09, 129.06, 127.41, 127.26, 58.15, 55.43, 47.73, 38.32, 21.60, 12.84, 12.48.

(2R)-2-((4-Methylphenyl)sulfonamido)-N-(2-oxocyclobutyl)-3-phenylpropanamide (6)

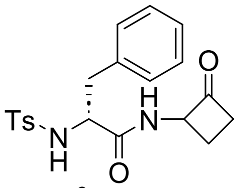

6

The *N*-Ts-carboxylic intermediate **12c** (50.0 mg, 0.129 mmol) was used in the reaction. The crude mixture of **6** was purified by column chromatography eluting with a gradient of ethyl acetate/hexane (20/80) and ethyl acetate/hexane (30/70) to afford compound **6** (18.1 mg, 36.2%) as white solid: mp 190-192 °C. ^{1}H NMR (500 MHz, $CDCl_3$) δ 7.57 – 7.48 (m, 2H), 7.24 – 7.14 (m, 5H), 6.95 – 6.86 (m, 2H), 6.75 (NH, d, *J* = 8.3 Hz, 1H), 4.98 (dtt, *J* = 10.2, 8.3, 1.9 Hz, 0.5H), 4.87 (NH, d, *J* = 6.6 Hz, 0.5H), 4.82 (NH, d, *J* = 6.8 Hz, 0.5H), 4.78 – 4.68 (m, 0.5H), 3.87 (dtd, *J* = 16.8, 7.0, 6.1 Hz, 1H), 3.07 – 2.93 (m, 1.5H), 2.96 – 2.91 (m, 1H), 2.93 – 2.85 (m, 1H), 2.83 (dd, *J* = 14.1, 5.8 Hz, 0.5H), 2.44 (s, 1.5H), 2.43 (s, 1.5H), 2.43 – 2.36 (m, 0.5H), 2.39 – 2.31 (m, 0.5H), 2.16 (dddd, *J* = 11.2, 10.3, 8.9, 8.0 Hz, 0.5H), 1.86 (dtd, *J* = 11.2, 9.7, 8.4 Hz, 0.5H) ^{13}C NMR (126 MHz, $CDCl_3$) δ 204.49, 204.40, 170.23, 169.93, 144.15, 144.09, 135.42, 135.30, 134.92, 134.71, 129.95, 129.90, 129.26, 129.14, 129.04, 127.42,

127.39, 127.21, 127.19, 77.27, 77.02, 76.77, 64.30, 63.68, 57.62, 57.29, 42.33, 42.13, 38.19, 37.74, 21.61, 21.59, 19.43, 18.90.

Benzyl ((2S)-3-(4-hydroxyphenyl)-1-oxo-1-((2-oxocyclobutyl)amino)propan-2-yl)carbamate (7)

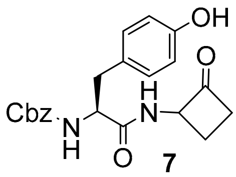

7

The crude mixture of **7** was purified by column chromatography eluting with a gradient of ethyl acetate/hexane (20/80) to (30/70) Combined fractions were recrystallized from hot methylene chloride to afford compound **7** (14.6 mg, 24%) as small needle-like crystals: mp 154-155 °C. ^{1}H NMR (500 MHz, acetonitrile-d_3) δ 7.42 – 7.26 (m, 4H), 7.23 – 7.13 (m, 1H), 7.11 – 7.04 (m, 2H), 6.77 – 6.71 (m, 2H), 5.88 (dd, J = 12.9, 8.5 Hz, 1H), 5.08 (d, J = 12.8 Hz, 1H), 4.99 (d, J = 12.7 Hz, 1H), 4.80 (dddd, J = 20.0, 10.0, 5.9, 2.1 Hz, 1H), 4.25 (dtd, J = 17.5, 8.7, 5.3 Hz, 1H), 3.04 (ddd, J = 13.6, 7.8, 5.4 Hz, 1H), 2.96 – 2.81 (m, 1H), 2.79 (dddd, J = 14.1, 12.0, 8.3, 2.7 Hz, 1H), 2.15 – 1.91 (m, 1H) ^{13}C NMR (126 MHz, CD_3CN) δ 206.08, 171.17, 171.10, 155.73, 137.13, 130.43, 128.45, 128.27, 128.22, 127.86, 127.51, 115.09, 66.07, 63.71, 63.63, 56.31, 56.14, 41.39, 41.37, 18.33, 18.14.

(2R)-2-((4-Methoxyphenyl)sulfonamido)-3-methyl-*N*-(2-oxocyclobutyl)butanamide (8)

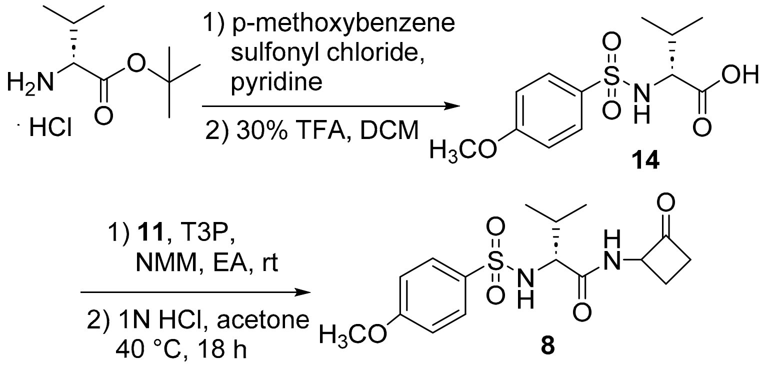

D-Valine tert-butyl ester HCl salt (was dissolved in anhydrous pyridine and stirred until all of the solutes were dissolved. The reaction mixture was cooled to 0 °C with an ice bath, and 4-methoxybenzenesulfonyl chloride (1.45 mmol) was added to the stirring reaction mixture. Then the reaction was allowed to warm up to rt and stirred at rt for 24 h with periodic monitoring using HLPC. Upon completion, the reaction mixture was diluted with methylene chloride (3 mL) and the organic layer was washed successively with water (3 mL), 1N HCl (10 x 3 mL), and brine (3 mL) and then dried over Na_2SO_4. The solvent was removed by evaporation under reduced pressure providing a pale-yellow solid. Without further purification p-methoxyphenylfulfonyl-D-valine tert butyl ester **14** was subjected to hydrolysis. To a solution of **14** (64.7 mg, 0.188 mmol) in methylene chloride (0.94 mL), TFA (282 µL, 30% v/v) was added under nitrogen. The mixture was stirred at room temperature overnight and monitored by TLC (EA/hexane = 50/50) The solvent was evaporated under vacuum. To the resultant residue toluene (2 mL) was added to enable the formation of an azeotrope. The excess TFA was removed by evaporation on a rotary evaporator at 50°C providing **14** as a yellow crystalline solid (52.6 mg, 97%) HPLC purity of the product **14** was 97%. Proton and carbon NMR spectra of the ((4-

methoxyphenyl)sulfonyl)-D-valine (**14**) matched the published data.[3] Compound **8** was synthesized from intermediate **14** (30 mg, 0.104 mmol) following the general procedure of the T3P coupling reactions of *N*-Ts amino acids with 2-aminocyclobutanone synthon **11** and the acetal intermediate was subjected to hydrolysis using general reaction conditions (1 M HCl, acetone, H_2O) and stirred at 40 °C for 18 hours. The crude mixture of **8** was purified by column chromatography using ethyl acetate/hexane (60/40) to afford p-methoxyphenylsulfonyl-D-valine cyclobutanone analog **8** (15.5 mg, 42%) as a white solid: mp 179-181. ^{1}H NMR (500 MHz, $CDCl_3$) δ 7.84 – 7.77 (m, 2H), 7.04 – 6.96 (m, 2H), 6.51 (dd, *J* = 16.8, 7.7 Hz, 1H), 5.08 (dd, *J* = 13.7, 7.8 Hz, 1H), 4.89 – 4.76 (m, 1H), 3.90 (s,1.5H), 3.89 (s, 1.5H), 3.48 (ddd, *J* = 16.5, 7.8, 5.1 Hz, 1H), 2.99 – 2.87 (m, 2H), 2.43 – 2.30 (m, 1H), 2.17 – 2.06 (m, 1H), 2.01 – 1.94 (m, 0.5H), 1.87 (dtd, *J* = 11.0, 9.6, 8.1 Hz, 0.5H),, 0.87 (dd, *J* = 6.9, 3.2 Hz, 3H), 0.81 (t, *J* = 6.7 Hz, 3H) ^{13}C NMR (126 MHz, $CDCl_3$) δ 204.22, 170.37, 170.30, 163.28, 130.53, 129.59, 114.37, 64.08, 63.90, 61.82, 61.80, 55.68, 42.29, 42.21, 31.11, 31.05, 19.19, 19.04, 17.15, 17.12. HRMS (ESI): Calcd for (MH^+) $C_{16}H_{23}N_2O_5S$: 355.1322, found 355.1309.

Benzyl ((2*S*)-1-oxo-1-((2-oxocyclobutyl)amino)-3-phenylpropan-2-yl)carbamate (9)

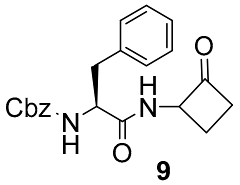

9

The crude mixture of **9** was purified by column chromatography eluting with a gradient of ethyl acetate/hexane (20/80) to (30/70) Appropriate fractions were combined and concentrated to give white solid (94% HPLC purity) which was then recrystallized from hot methylene chloride to give a white needle-like crystals of compound **9** with 97% HPLC

purity (30.2 mg, 49%): mp 100-101 °C. ^{1}H NMR (500 MHz, $CDCl_3$) δ 7.40 – 7.23 (m, 8H), 7.23 – 7.17 (m, 2H), 6.63 (NH, d, *J* = 7.6 Hz, 0.5H), 6.48 (NH, d, *J* = 7.9 Hz, 0.5H), 5.46 (t, *J* = 10.6 Hz, 1H), 5.07 (t, *J* = 3.3 Hz, 2H), 4.96 – 4.87 (m, 0.5H), 4.78 (qt, *J* = 7.9, 1.4 Hz, 0.5H), 4.45 (d, *J* = 7.0 Hz, 1H), 3.09 (d, *J* = 6.7 Hz, 2H), 2.98 – 2.83 (m, 2H), 2.38 (dd, *J* = 10.9, 5.2 Hz, 0.5H), 2.33 (dd, *J* = 10.3, 4.7 Hz, 0.5H), 2.03 (q, *J* = 9.6 Hz, 0.5H), 1.90 – 1.73 (m, 0.5H) ^{13}C NMR (126 MHz, $CDCl_3$) δ 204.79, 204.76, 170.84, 170.69, 156.03, 136.23, 136.23, 136.08, 129.40, 129.38, 128.76, 128.58, 128.26, 128.01, 127.14, 67.16, 64.03, 63.72, 55.97, 55.75, 42.16, 42.06, 38.63, 38.43, 19.35, 19.13. HRMS (ESI): Calcd for (MH+) $C_{21}H_{23}N_2O_4$: 367.1652, found 367.1646.

(S)-*N*-(2-hydroxy-2-methylpropyl)-2-((4-methylphenyl)sulfonamido)-3-phenylpropanamide (10)

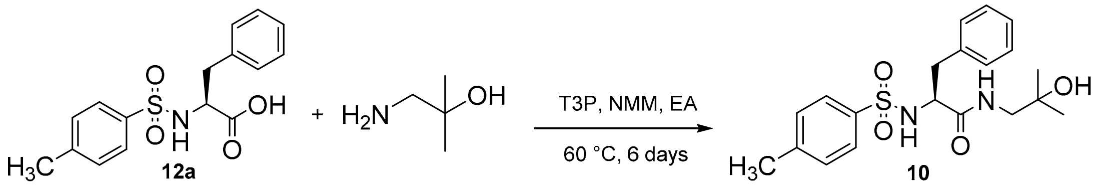

Ts-L-phenylalanine **12a** (50 mg, 0.129 mmol) was reacted with dimethylethanolamine (14.4 µL, 0.155 mmol) following the general T3P coupling procedure and stirred at 50 to 60 °C for 6 days until completion. The crude product (oil) of **10** was crystallized from hot EA/hexane = 20/80. When the EA/hexane solution was cooled to rt the product became an oil. Therefore, it is important to maintain the temperature of the solution to stay between 50 to 60 °C to allow a slow crystallization of the final product. Recrystallization provided compound **10** (29 mg, 58%) as clear needle-like crystals: mp 131-133 °C. ^{1}H NMR (500 MHz, $CDCl_3$) δ 7.53 – 7.47 (m, 2H), 7.23 – 7.14 (m, 5H), 6.96 – 6.90 (m, 2H), 6.74 (s, 1H), 4.93 (d, *J* = 6.2 Hz, 1H), 3.84 (dt, *J* = 7.8, 6.1 Hz, 1H), 3.29 (dd, *J* = 13.7, 6.9 Hz, 1H), 3.12 (dd, *J*

= 13.7, 5.5 Hz, 1H), 2.97 (dd, *J* = 14.0, 5.9 Hz, 1H), 2.91 (dd, *J* = 14.0, 7.7 Hz, 1H), 2.43 (s, 3H), 1.16 (d, *J* = 2.2 Hz, 6H) ^{13}C NMR (126 MHz, $CDCl_3$) δ 170.92, 144.05, 135.19, 135.16, 129.87, 129.09, 129.04, 127.37, 127.23, 70.85, 58.06, 50.30, 38.29, 27.17, 27.10, 21.58. HRMS (ESI): Calcd for (MH^+) $C_{20}H_{27}N_2O_4S$: 391.1686, found 391.1680.

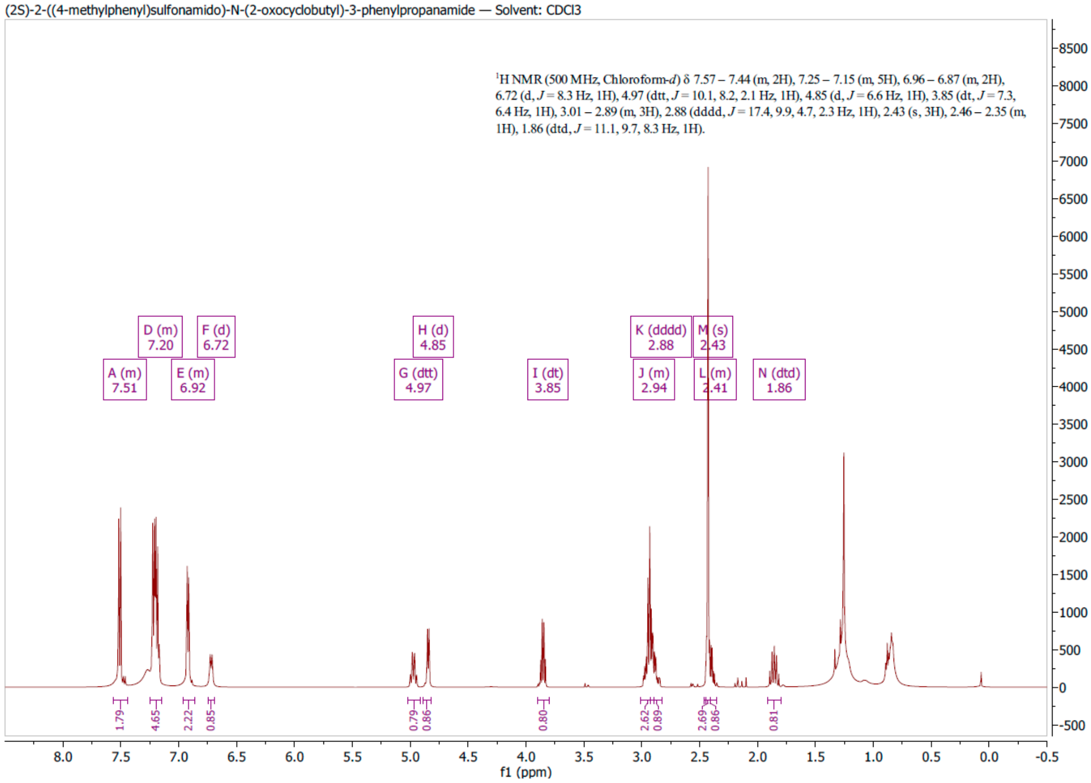

Figure S1: ^{1}H NMR spectrum of (2*S*)-2-((4-methylphenyl)sulfonamido)-*N*-(2-oxocyclobutyl)-3-phenylpropanamide (**1**)

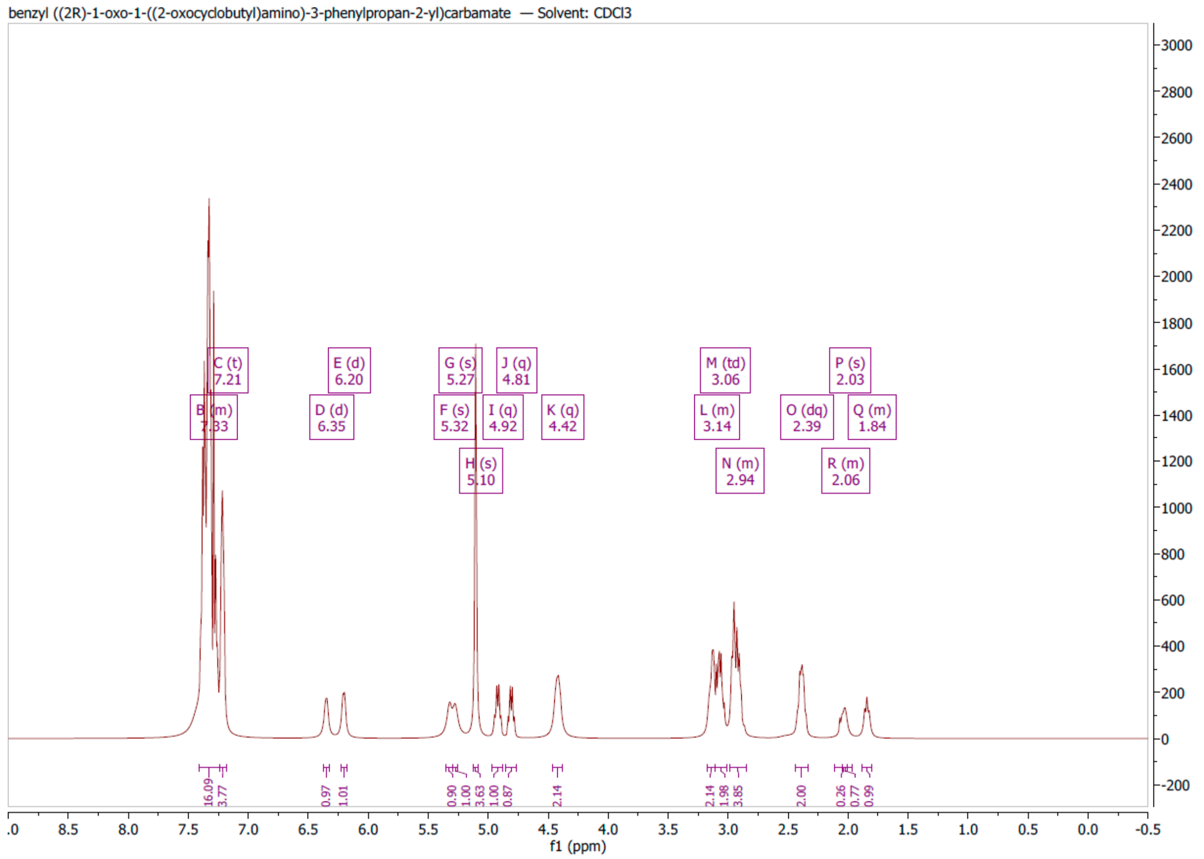

Figure S2: ^{1}H NMR spectrum of benzyl ((2R)-1-oxo-1-((2-oxocyclobutyl)amino)-3-phenylpropan-2-yl)carbamate (**2**)

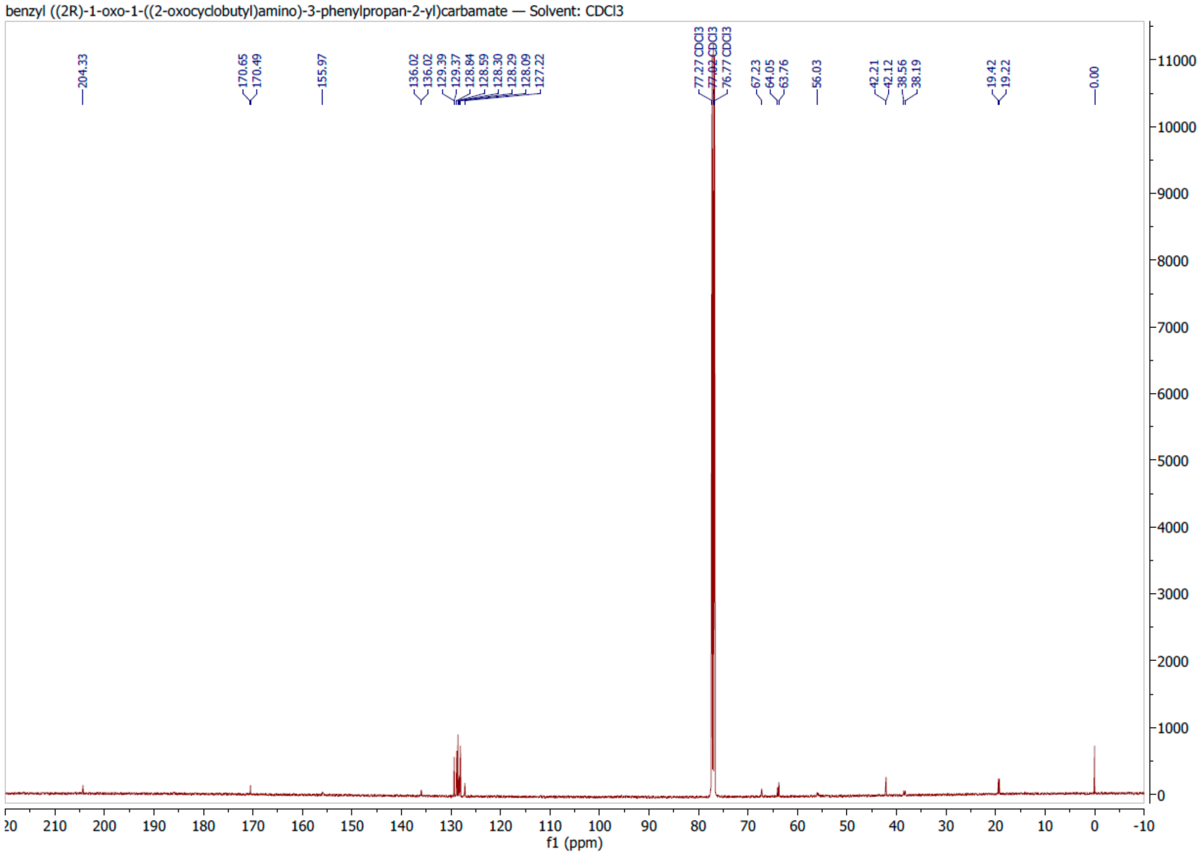

Figure S3: ^13^C spectrum of benzyl ((2R)-1-oxo-1-((2-oxocyclobutyl)amino)-3-phenylpropan-2-yl)carbamate (**2**)

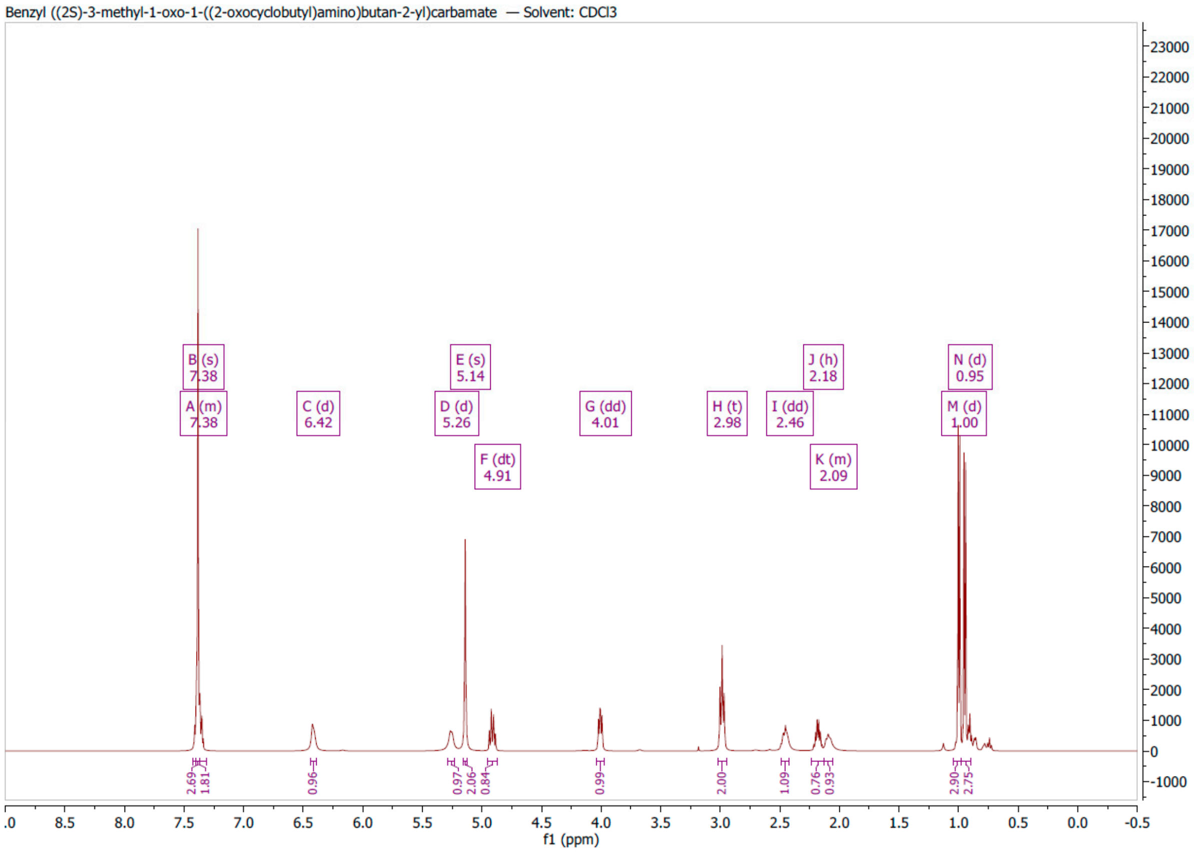

Figure S4: ^{1}H NMR spectrum of benzyl ((2S)-3-methyl-1-oxo-1-((2-oxocyclobutyl)amino)butan-2-yl)carbamate (**3**)

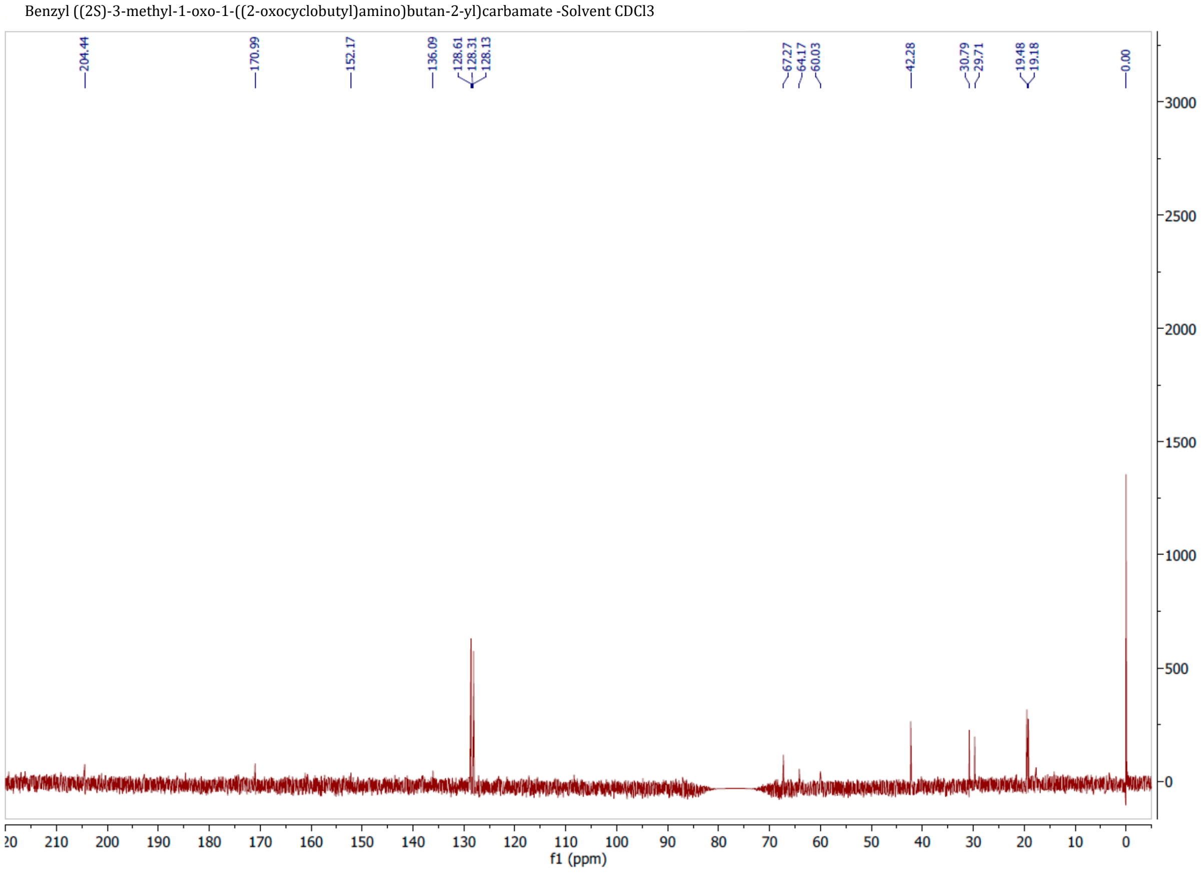

Figure S5: ^{13}C NMR spectrum of benzyl ((2S)-3-methyl-1-oxo-1-((2-oxocyclobutyl)amino)butan-2-yl)carbamate (**3**)

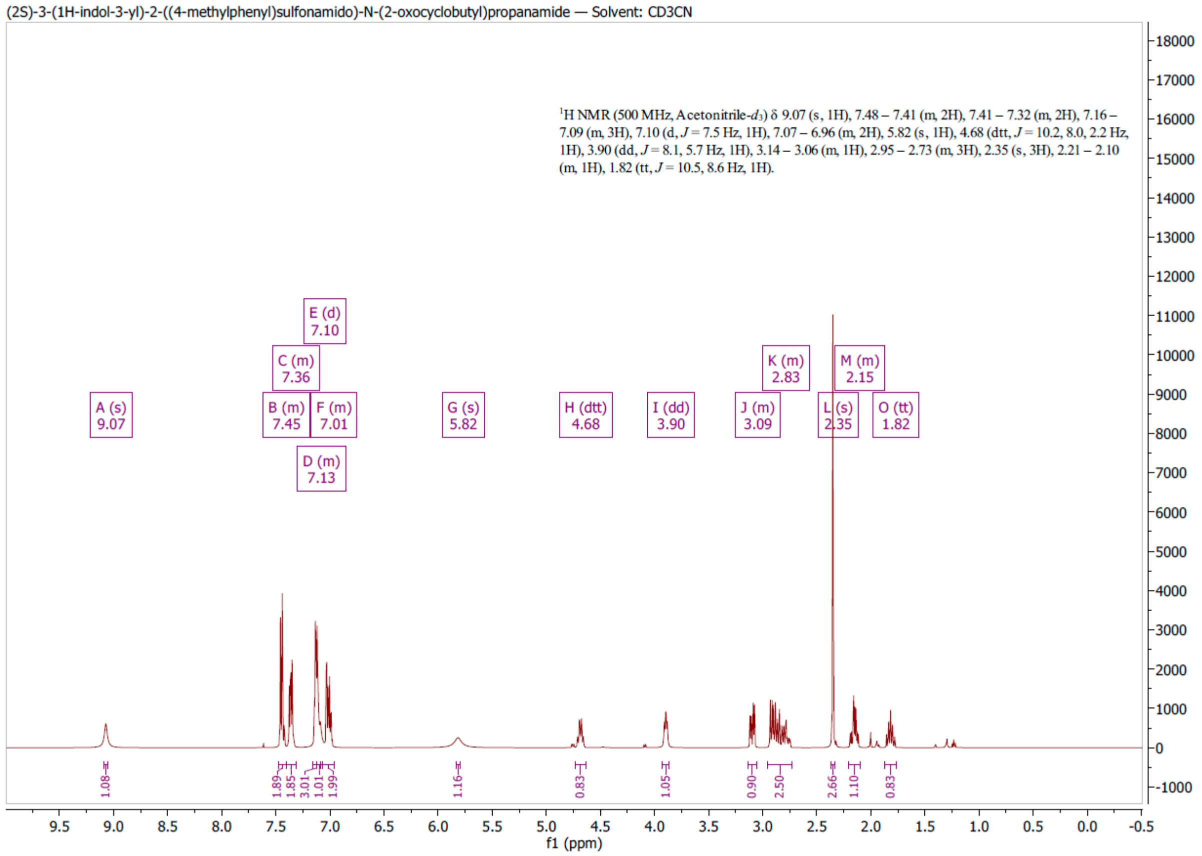

Figure S6: ¹H spectrum of (2*S*)-3-(1*H*-indol-3-yl)-2-((4-methylphenyl)sulfonamido)-*N*-(2-oxocyclobutyl)propanamide (**4**)

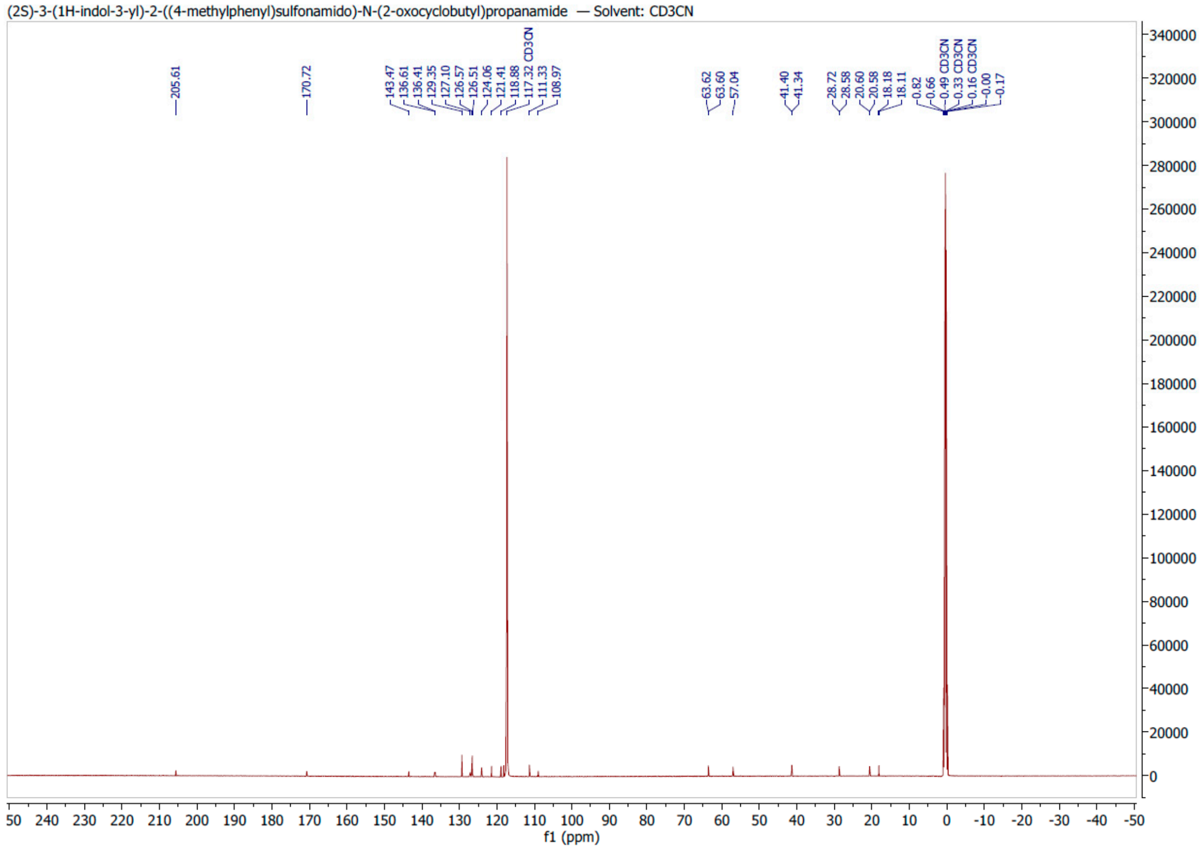

Figure S7: ^{13}C spectrum of (2*S*)-3-(1*H*-indol-3-yl)-2-((4-methylphenyl)sulfonamido)-*N*-(2-oxocyclobutyl)propanamide (**4**)

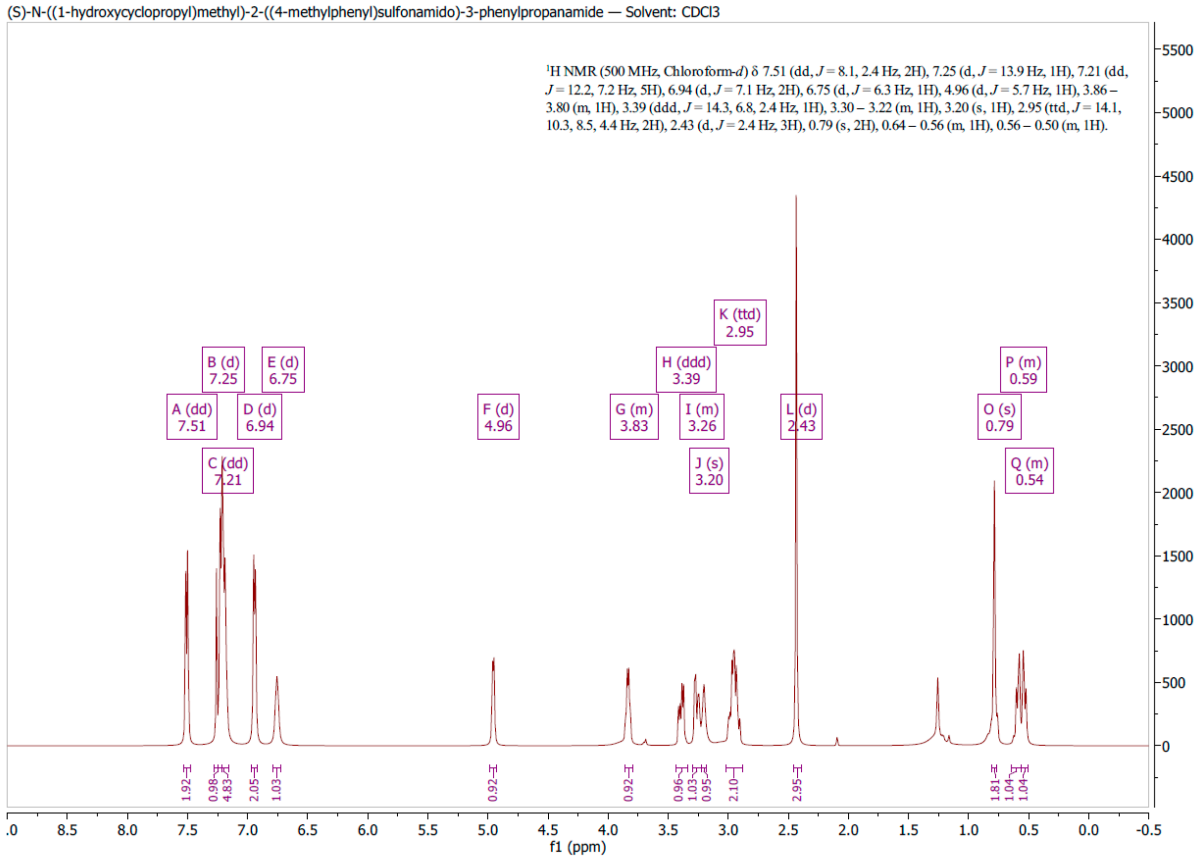

Figure S8: ^{1}H spectrum of (*S*)-*N*-((1-hydroxycyclopropyl)methyl)-2-((4-methylphenyl)sulfonamido)-3-phenylpropanamide (**5**)

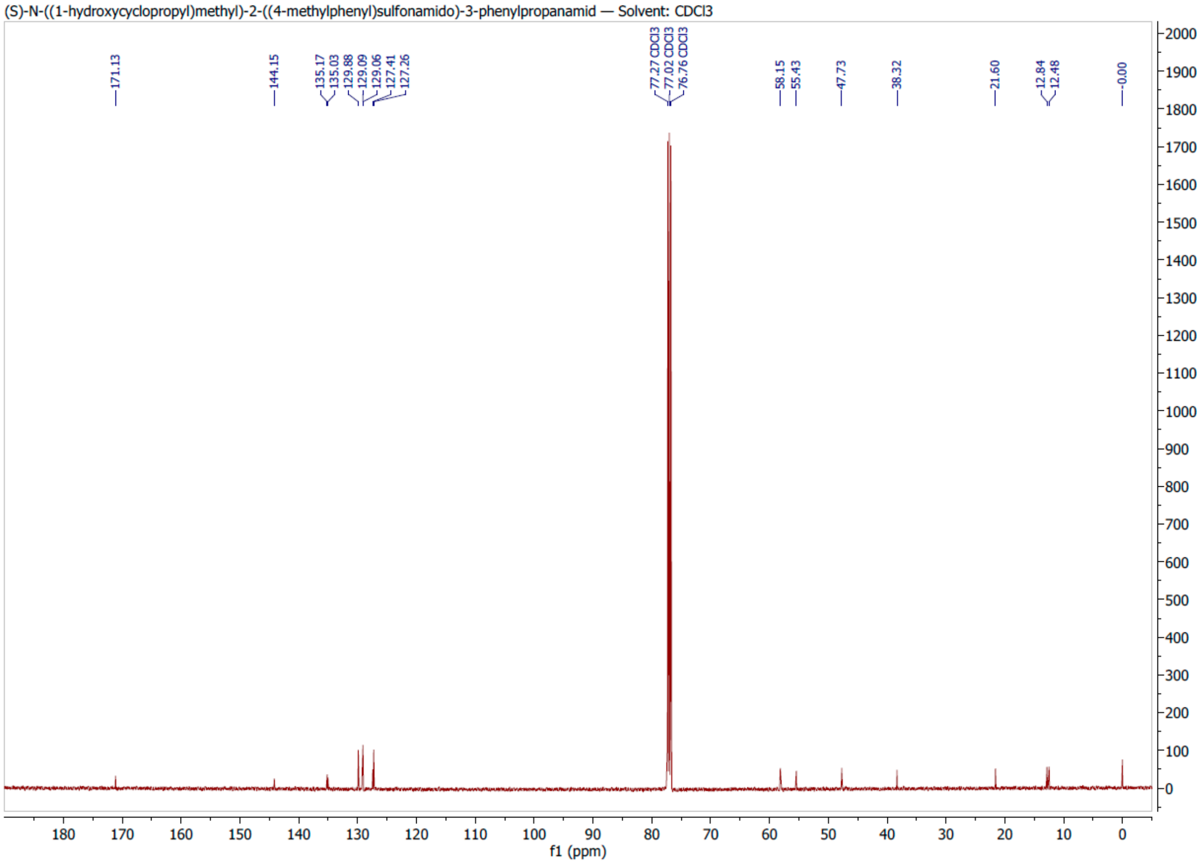

Figure S9: ^{13}C NMR spectrum of (*S*)-*N*-((1-hydroxycyclopropyl)methyl)-2-((4-methylphenyl)sulfonamido)-3-phenylpropanamide (**5**)

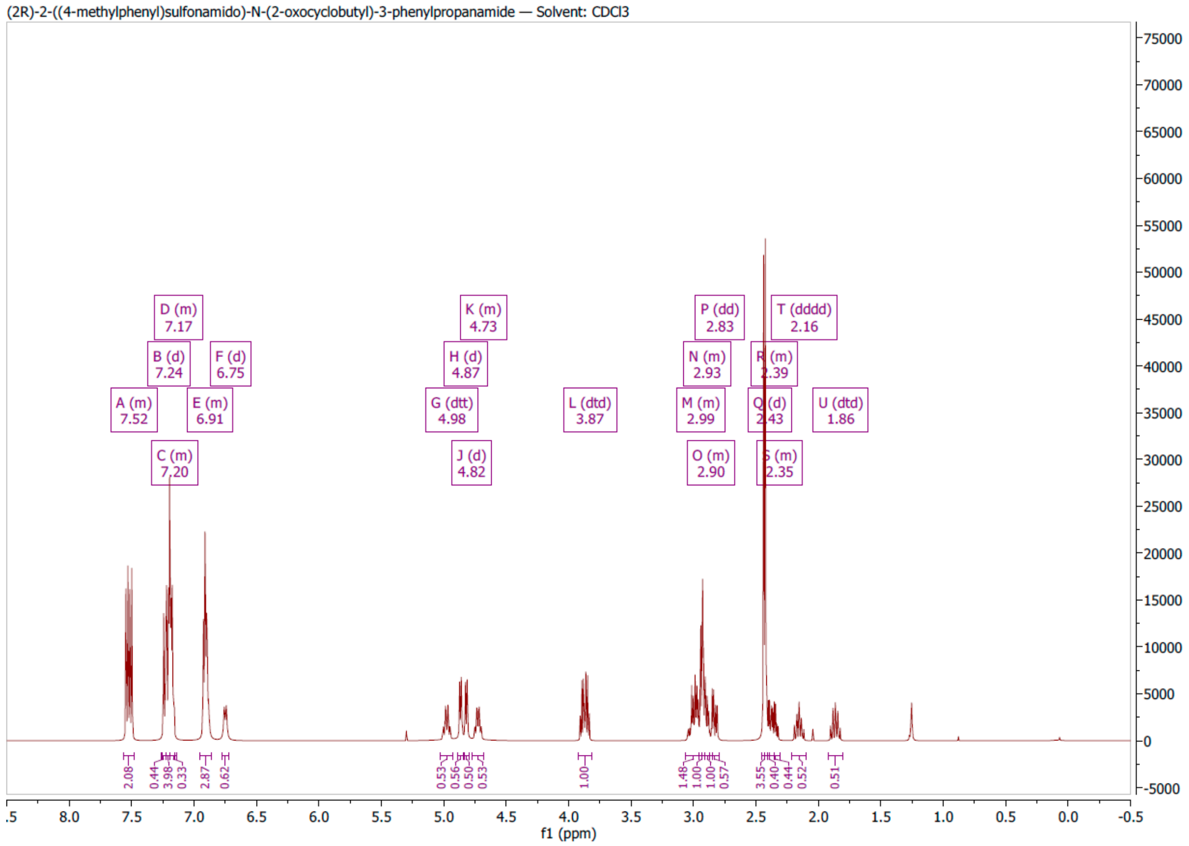

Figure S10: ^{1}H NMR spectrum of (2R)-2-((4-methylphenyl)sulfonamido)-*N*-(2-oxocyclobutyl)-3-phenylpropanamide (**6**)

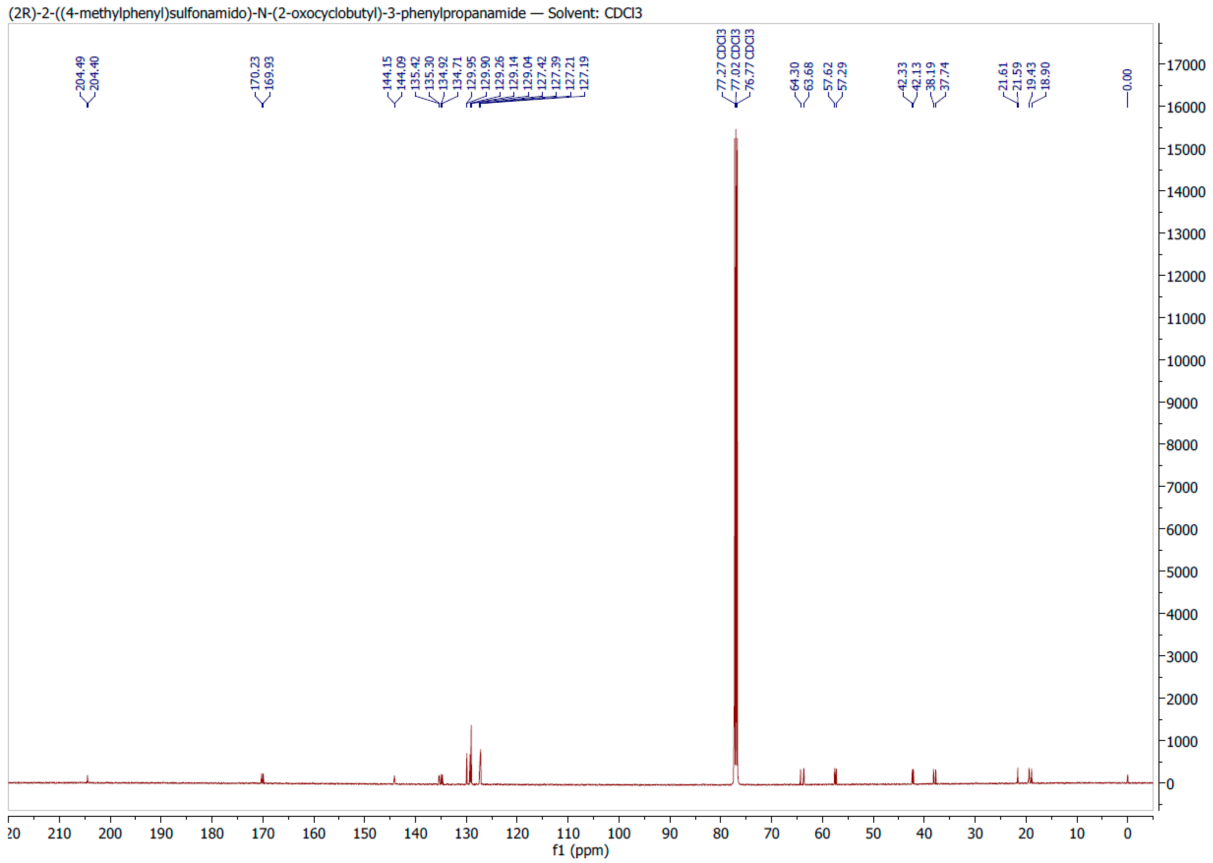

Figure S11: ^{13}C spectrum of (2R)-2-((4-methylphenyl)sulfonamido)-*N*-(2-oxocyclobutyl)-3-phenylpropanamide (**6**)

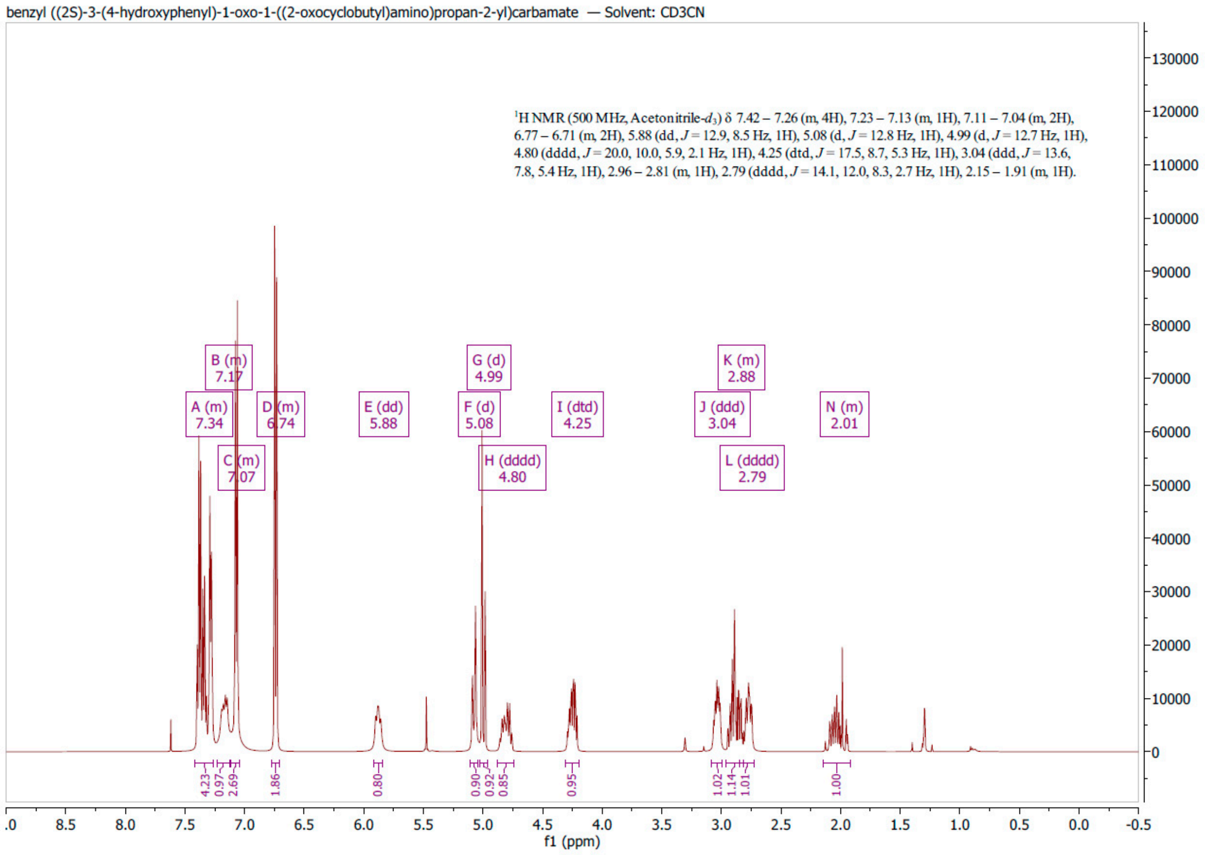

Figure S12: ^{1}H NMR spectrum of benzyl ((2S)-3-(4-hydroxyphenyl)-1-oxo-1-((2-oxocyclobutyl)amino)propan-2-yl)carbamate (**7**)

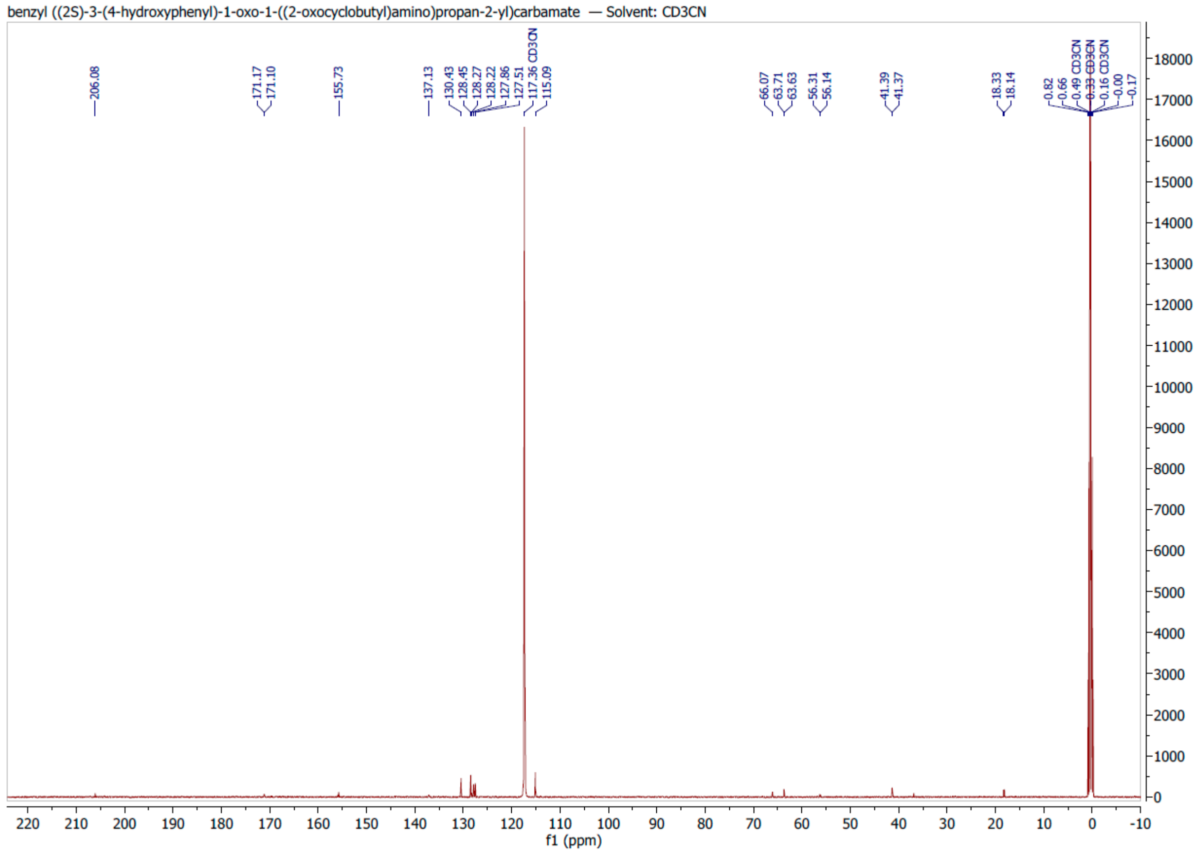

Figure S13: ^{13}C NMR spectrum of benzyl ((2S)-3-(4-hydroxyphenyl)-1-oxo-1-((2-oxocyclobutyl)amino)propan-2-yl)carbamate (**7**)

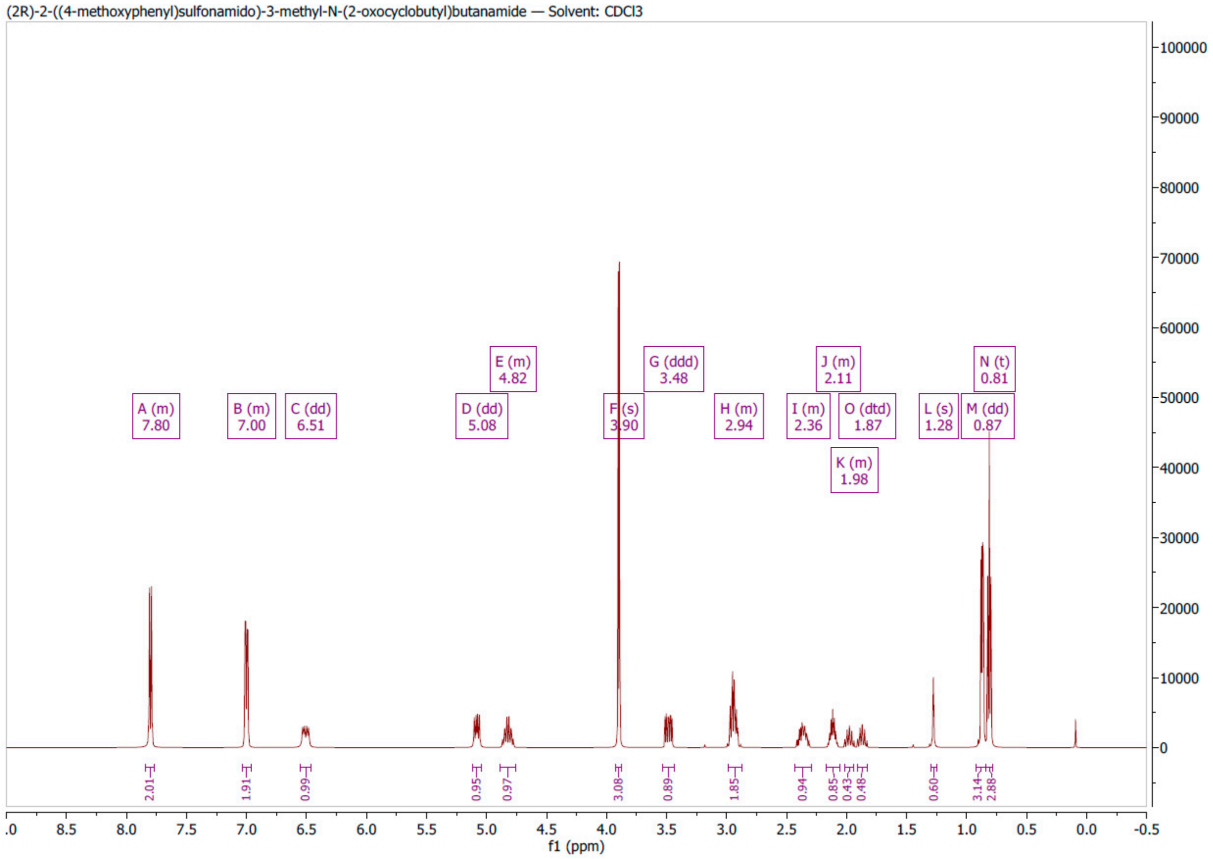

Figure S14: ^{1}H spectrum of (2R)-2-((4-methoxyphenyl)sulfonamido)-3-methyl-*N*-(2-oxocyclobutyl)butanamide (**8**)

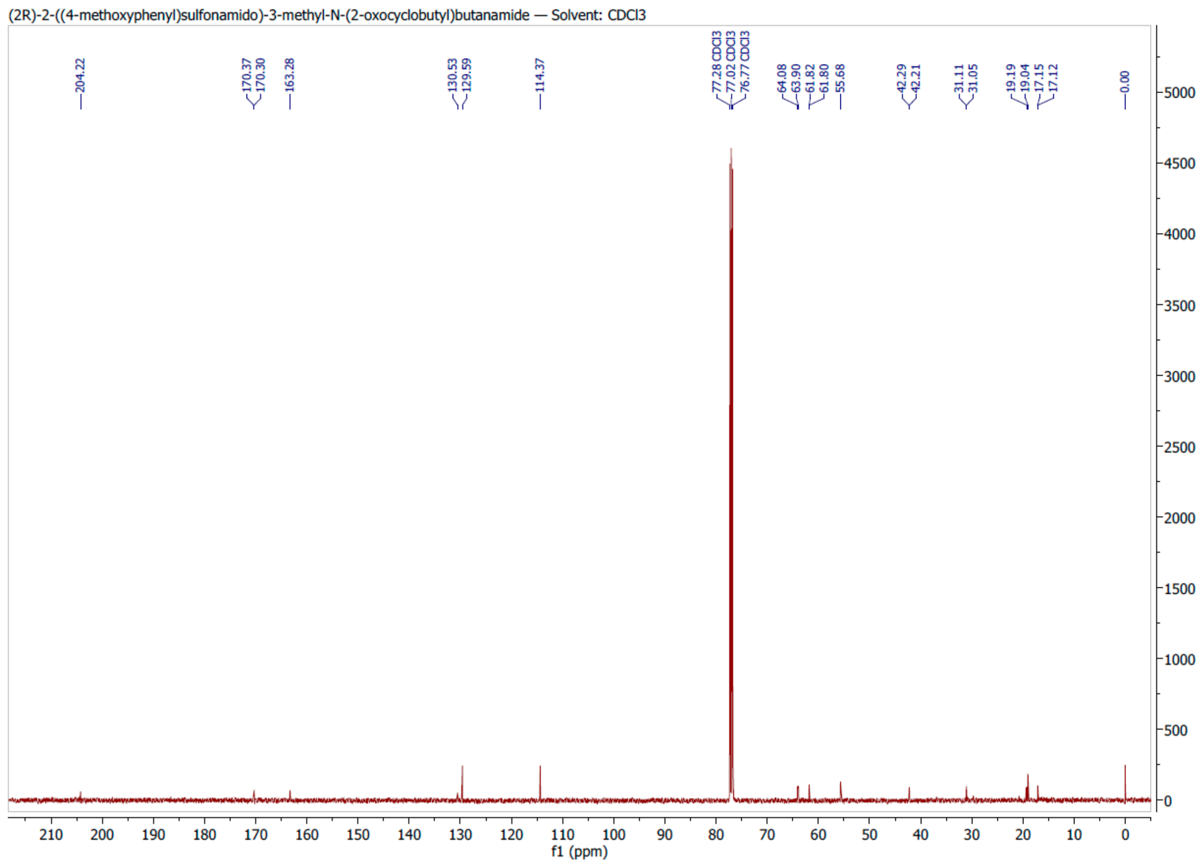

Figure S15: ^{13}C spectrum of (2R)-2-((4-methoxyphenyl)sulfonamido)-3-methyl-*N*-(2-oxocyclobutyl)butanamide (**8**)

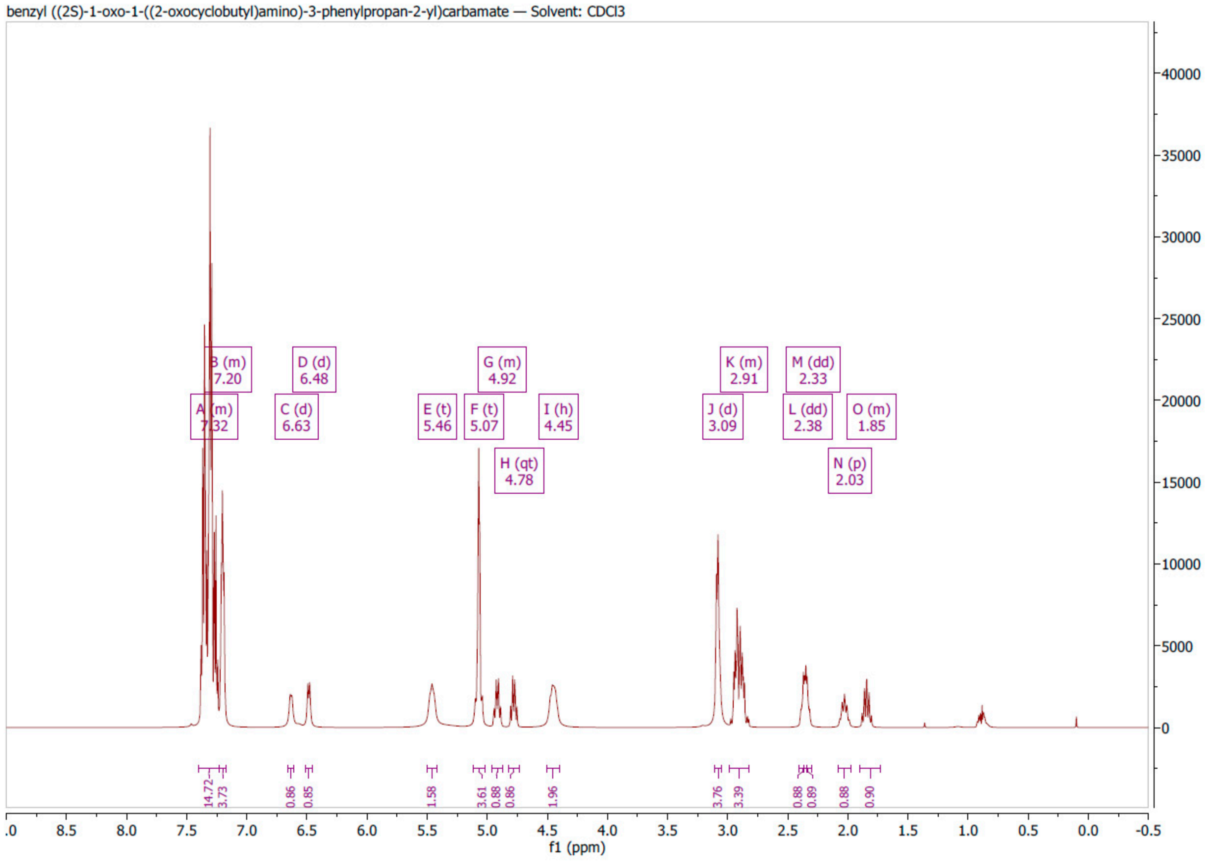

Figure S16: ^{1}H NMR spectrum of benzyl ((2S)-1-oxo-1-((2-oxocyclobutyl)amino)-3-phenylpropan-2-yl)carbamate (**9**)

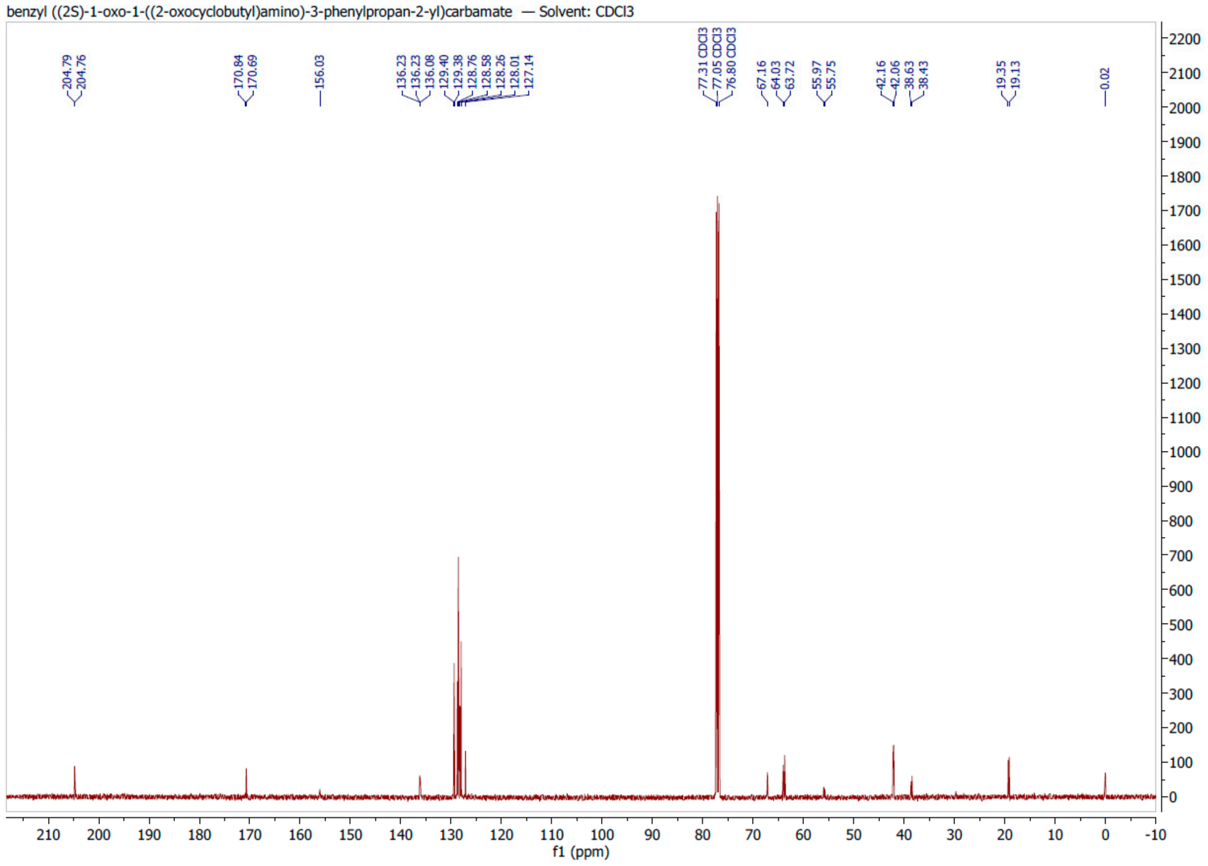

Figure S17: ^{13}C NMR spectrum of benzyl ((2*S*)-1-oxo-1-((2-oxocyclobutyl)amino)-3-phenylpropan-2-yl)carbamate (**9**)

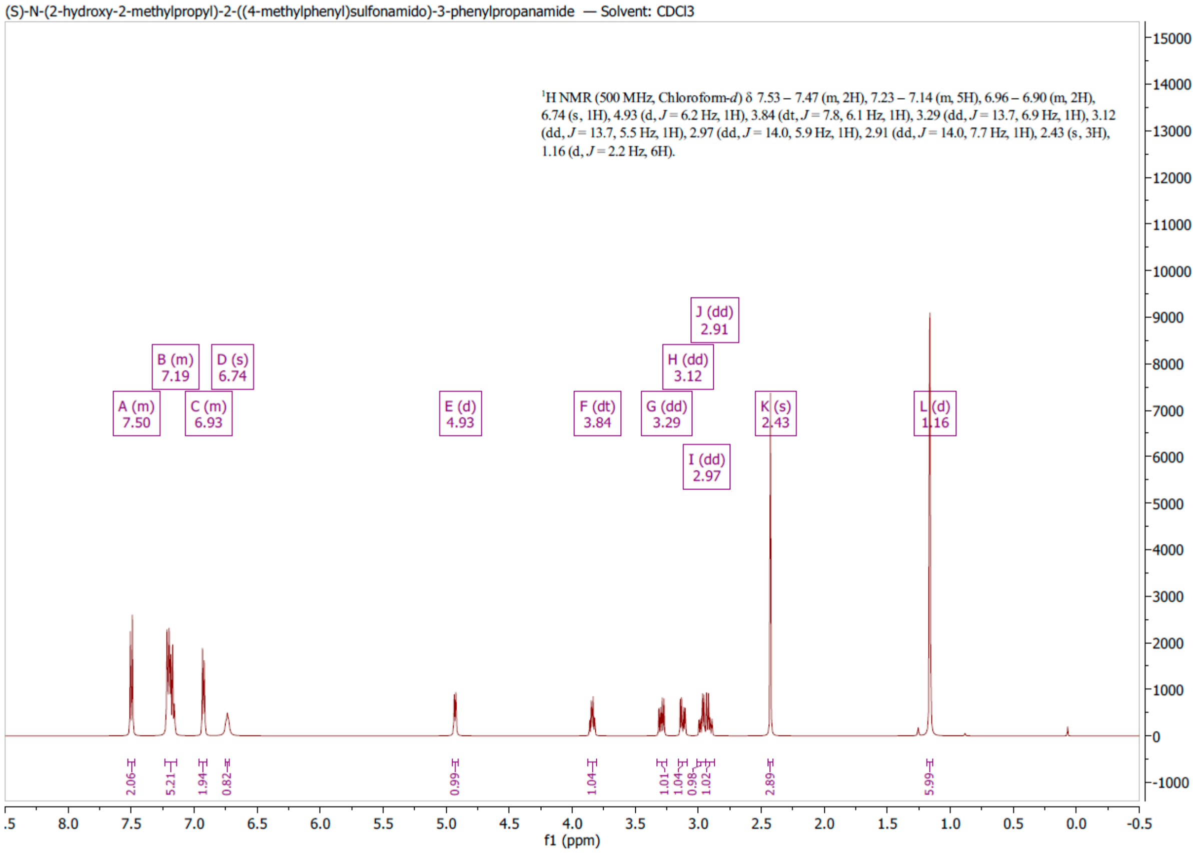

Figure S18: ^{1}H NMR spectrum of (S)-*N*-(2-hydroxy-2-methylpropyl)-2-((4-methylphenyl)sulfonamido)-3-phenylpropanamide (**10**)

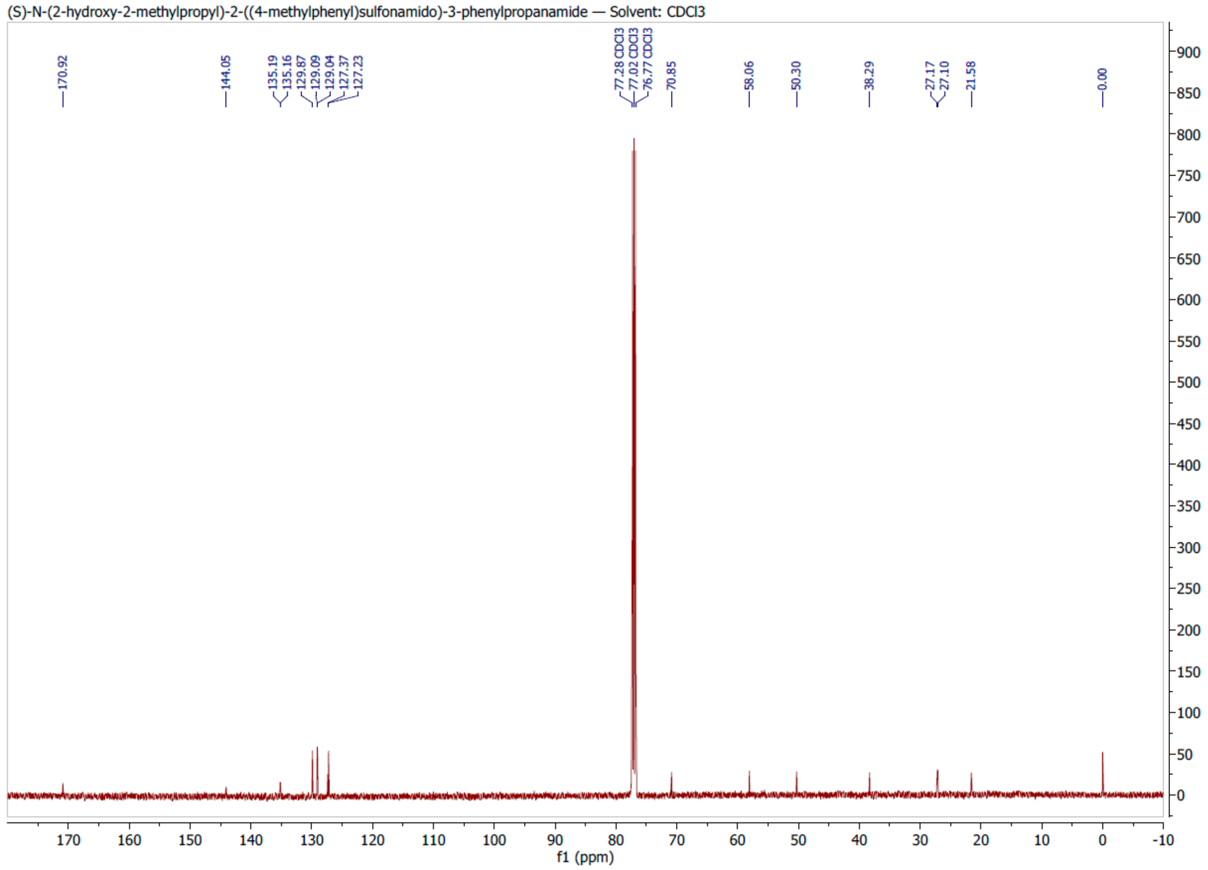

Figure S19: ^{13}C NMR spectrum of (*S*)-*N*-(2-hydroxy-2-methylpropyl)-2-((4-methylphenyl)sulfonamido)-3-phenylpropanamide (**10**)
